# Composite Objective Optimization of Finger Length Under Performance Trade-Offs and Constraints

**DOI:** 10.3390/biomimetics11080529

**Published:** 2026-07-30

**Authors:** Lei Jiang, Kaixin Lan, Xianwei Liu, Chaojie Fu, Yongbin Jin, Hongtao Wang

**Affiliations:** 1 Center for X-Mechanics, Zhejiang University, Hangzhou 310012, China; 12524001@zju.edu.cn (L.J.); 22424122@zju.edu.cn (K.L.); 3140104175@zju.edu.cn (X.L.); 12124058@zju.edu.cn (C.F.); 2 State Key Laboratory of Fluid Power and Mechatronic System, Zhejiang University, Hangzhou 310012, China; 3 ZJU-Hangzhou Global Scientific and Technological Innovation Center, Zhejiang University, Hangzhou 311200, China

**Keywords:** biomimetic finger, multi-objective optimization, phalanx length, Pareto optimality, workspace analysis, kinematic dexterity, key-press range

## Abstract

The inherent trade-off between structural compactness and functional versatility poses a fundamental challenge in biomimetic robotic hand design. This paper presents a multi-objective optimization framework for determining the optimal phalanx length allocation of a biomimetic finger under a fixed total-length constraint. Three performance criteria are formulated: grasp capability, measured by the area of a novel shared workspace (Region II) between power grasping and precision manipulation; kinematic dexterity, evaluated as the global average of the reciprocal condition number of the Jacobian matrix; and key-press range, defined as the maximum static fingertip span under perpendicularity and slope constraints. A full grid search reveals distinct optimal configurations for each objective. Pareto analysis of 117 non-dominated solutions shows that the key-press range is most sensitive to dimensional variations, with a 24.7% performance spread. A hierarchical selection strategy that prioritizes the key-press range while balancing the other two objectives yields a recommended compromise design. Experimental validation of the key-press reachability for the recommended compromise design achieves a 98.8% keystroke success rate over 1000 cross-row strikes without wrist movement. These results confirm its practical feasibility for fine manipulation tasks, while experimental characterization of the grasp capability and kinematic dexterity objectives remains as future work.

## 1. Introduction

The design of anthropomorphic robotic hands has attracted sustained attention in the field of biomimetics, driven by the goal of replicating the versatility and dexterity of the human hand [1,2,3,4,5,6]. However, a persistent challenge lies in reconciling structural compactness with functional performance. Achieving high degrees of freedom (DoF) often necessitates complex kinematic chains and additional actuators, which inevitably increase finger dimensions and mass [4,7,8,9]. In applications such as prosthetics, wearable robotics, and compact automation, the total finger length is typically constrained by anthropomorphic or spatial requirements. Under such constraints, the allocation of lengths among the three phalanges becomes a critical design variable that directly influences grasp stability, manipulation dexterity, and task-specific reachability [10,11].

The human hand itself embodies an elegant solution to this trade-off. Its phalangeal proportions—with a relatively long proximal phalanx, an intermediate middle phalanx, and a shorter distal phalanx—have evolved to support a wide spectrum of functions, from powerful enveloping grasps to precise fingertip manipulations [11,12,13,14]. This biological precedent suggests that biomimetic finger design should likewise pursue a balanced optimization across multiple performance criteria rather than maximizing any single metric in isolation.

Prior research has established various individual metrics for robotic finger evaluation and optimization. Workspace volume and manipulability ellipsoids have been widely used to characterize kinematic reachability and dexterity [15,16,17,18]. Force-closure conditions and grasp quality measures have been developed to quantify grasp stability [19,20,21,22,23]. The condition number of the Jacobian matrix has been adopted as an isotropy index, with values closer to unity indicating more uniform force and motion transmission [16,24,25]. However, these criteria have typically been applied in isolation, and a unified framework that simultaneously accounts for grasping capability (encompassing both power and precision modes), kinematic dexterity, and task-specific reachability remains absent from the literature. Furthermore, existing optimization studies often treat phalanx lengths as independent variables without explicitly enforcing a total-length constraint [26,27,28,29,30,31,32], which limits their practical applicability in size-constrained designs. The trade-offs among competing objectives have rarely been systematically quantified, leaving designers without clear guidance on how to prioritize different performance aspects according to application scenarios.

To address these gaps, this paper presents a multi-objective optimization framework for determining the optimal phalanx lengths of a biomimetic finger under a fixed total-length constraint. Three physically meaningful objective functions are established: $f_{1}$ (grasp capability) quantifies the area of a shared workspace region (Region II) that is common to both power grasping and precision manipulation; $f_{2}$ (kinematic dexterity) represents the global average of the reciprocal condition number over the joint space; and $f_{3}$ (key-press range) measures the maximum static fingertip span on a key plane under perpendicularity and slope constraints. A full grid search over the constrained design space is performed to maximize each objective individually, revealing distinct optimal configurations. A Pareto-based analysis [33] identifies the inherent trade-offs, with the key-press range being the most sensitive to dimensional variations. A hierarchical selection strategy is then adopted: solutions that achieve at least 95% of the maximum $f_{3}$ are retained, and within this subset, the normalized sum of $f_{1}$ and $f_{2}$ is maximized to obtain a single recommended compromise design. The main contributions of this work are threefold:

1. **Workspace shared-region modeling.** The fingertip workspace is divided into five functional regions based on geometric criteria. Region II, the unique area shared by power grasp and precision manipulation postures, is identified, and its area is derived analytically via sector–triangle decomposition.
2. **Parametric analysis of the condition number.** The reciprocal condition number of the Jacobian matrix is averaged over 1000 uniformly sampled postures to evaluate global dexterity. Parametric sweeps reveal the influence of each phalanx length on dexterity, with the optimum located on the feasible domain boundary.
3. **Single-objective prototype verification.** A physical prototype built with the recommended compromise design is experimentally tested in a keyboard typing task, achieving a 98.8% success rate over 1000 cross-row key strikes without wrist movement, confirming practical feasibility.

Scope and limitations of the present validation. A physical prototype built with the recommended compromise dimensions ($l_{1}=40.44$ mm, $l_{2}=21.39$ mm, $l_{3}=19.17$ mm) is experimentally tested in a keyboard typing task. The prototype achieves a stable keystroke frequency of approximately 5.5 Hz and an overall success rate of 98.8% across 1000 cross-row key strikes without wrist movement, confirming the practical viability of the optimized design. While the experimental validation is limited to the recommended compromise design (rather than multiple points on the Pareto front or different weight combinations), the complete optimization framework—including single-objective maxima, Pareto front, correlation analysis, and hierarchical selection—is fully established analytically and through simulation. Multi-objective experimental verification of other Pareto points is left for future work. Furthermore, the experimental validation focuses exclusively on the key-press reachability ($f_{3}$); physical testing of the grasp capability ($f_{1}$) and kinematic dexterity ($f_{2}$) has not yet been performed and is deferred to future work.

The remainder of this paper is organized as follows. Section 2 describes the materials and methods, including the constrained design space, the three objective functions, the Pareto analysis, and the hierarchical selection strategy. Section 3 presents the optimization results for each objective, the Pareto front, and the experimental validation. Section 4 discusses the implications and limitations, and Section 5 concludes the paper.

## 2. Materials and Methods

### 2.1. Design Problem Formulation and Multi-Objective Optimization Framework

The design of anthropomorphic robotic hands inherently involves a fundamental tension between structural compactness and functional versatility. Under a fixed total finger length, the proportional allocation of the three phalanx lengths—proximal $l_{1}$, middle $l_{2}$, and distal $l_{3}$—exerts a direct and coupled influence on multiple aspects of performance, including grasping stability, kinematic dexterity, and fine manipulation reachability. Identifying the link length configuration that best reconciles these competing demands constitutes a nontrivial multi-objective design problem. This paper addresses the problem within a constrained design space defined by a constant total length $l_{1}+l_{2}+l_{3}=81$ mm and practical proportion bounds $l_{1}\in [40,44]$ mm, $l_{2}\in [19,23]$ mm, which together imply $l_{3}=81-l_{1}-l_{2}\in [16,20]$ mm.

**Figure 1:**
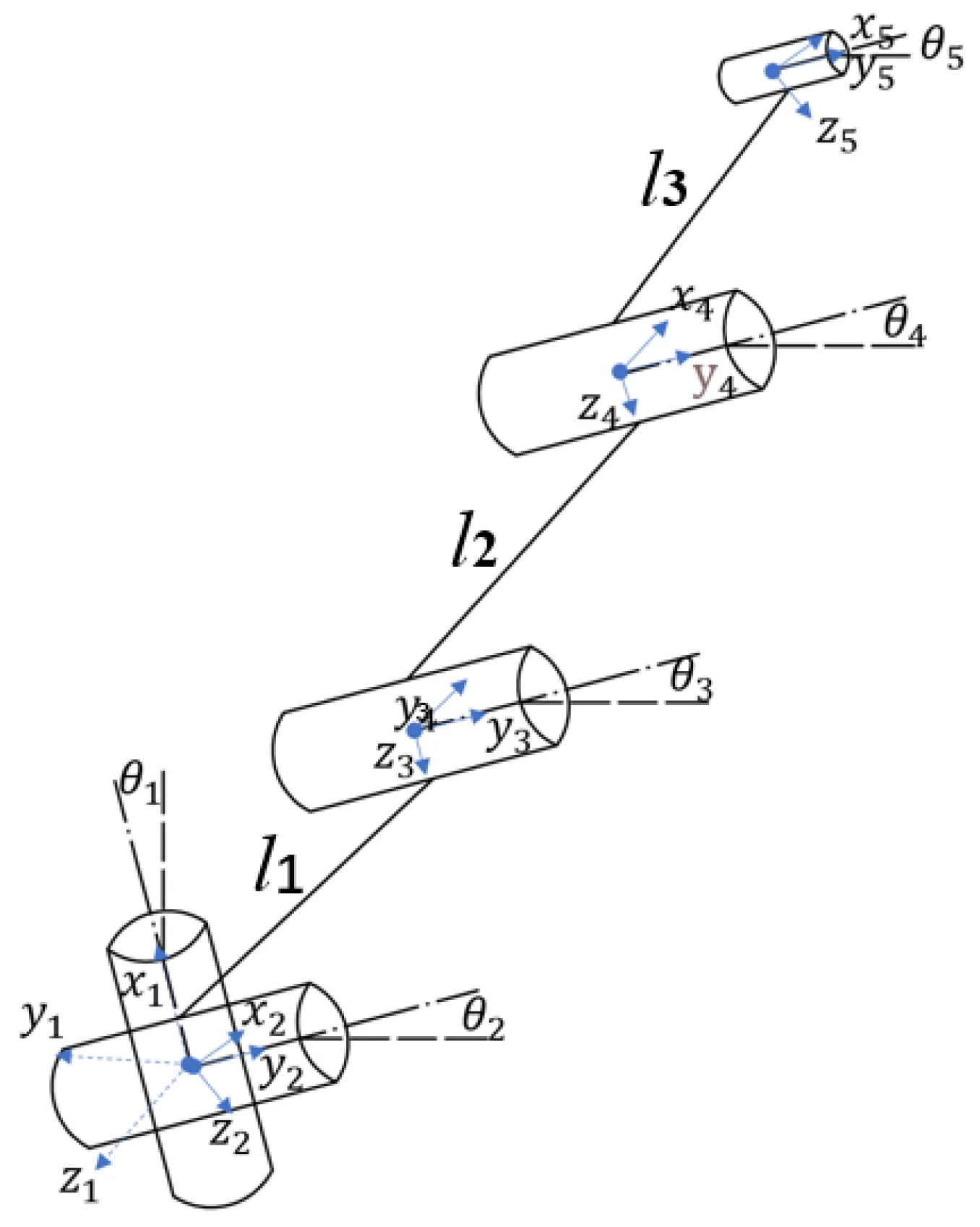
Simplified coordinate system of the dexterous hand.

To quantitatively analyze the kinematic performance, the finger is modeled as three rigid links with joint angles defined within the palmar flexion direction: $\theta_{2}$ for the proximal phalanx $l_{1}$, $\theta_{3}$ for the relative angle between $l_{2}$ and $l_{1}$, and $\theta_{4}$ for the relative angle between $l_{3}$ and $l_{2}$. All joint angles vary within the palmar flexion range: $\theta_{2}\in [0^{\circ },90^{\circ }]$, $\theta_{3}\in [0^{\circ },90^{\circ }]$, and $\theta_{4}\in [0^{\circ },90^{\circ }]$. The corresponding kinematic coordinate system is shown in Figure 1. In human hands, the distal interphalangeal (DIP) and proximal interphalangeal (PIP) joints exhibit a natural coupling during flexion, with coupling coefficients typically ranging from 0.6 to 1 for tasks such as piano playing [13,14,34]. Inspired by this biomechanical feature, many biomimetic finger designs adopt a mechanical coupling between $\theta_{3}$ and $\theta_{4}$ [13,14,35,36,37]. In this paper, a coupling coefficient of 1 is assumed for the analyses of $f_{1}$, $f_{2}$, and $f_{3}$ (Section 3.1, Section 3.2 and Section 3.3); a comparison with the case of κ = 0.7 is then provided in Section 3.6.

Three objective functions are established, each quantifying a distinct and practically relevant facet of hand capability. The first, the grasp-capability index $f_{1}$, is motivated by the observation that power grasping and precision manipulation favor different regions of the fingertip workspace. Through a geometric analysis of characteristic postures detailed in Section 2.2, the workspace is partitioned into five functional sub-regions, and a specific sub-region termed Region II is identified as the only one shared by both grasp modes. The area of Region II is therefore adopted as $f_{1}$, with a larger value indicating a broader posture set that simultaneously supports stable enveloping and accurate fingertip control, thereby capturing the grasp–manipulation compromise in a purely kinematic manner. The second objective, the kinematic dexterity index $f_{2}$, quantifies the isotropy of motion and force transmission at the fingertip via the condition number of the finger’s Jacobian matrix (Section 2.3). To reflect global performance rather than behavior at a single configuration, $f_{2}$ is defined as the average of the reciprocal condition number over 1000 postures uniformly sampled across the joint space; a larger $f_{2}$ corresponds to more uniform manipulability and a greater average margin from singular configurations. The third objective, the key-press range index $f_{3}$, captures the effective fingertip coverage on a key plane under a stationary wrist for fine manipulation tasks such as keyboard typing (Section 2.4). It is defined as the maximum straight-line span *MN* between the nearest and farthest reachable key points, subject to a perpendicularity constraint that prevents jamming between keycaps and a slope inequality that ensures geometric feasibility of the farthest reach. A larger $f_{3}$ directly expands the static key coverage and reduces the need for wrist movement.

These three objectives quantify distinct aspects of hand performance that are potentially competing rather than simultaneously attainable. Maximizing each objective in isolation drives the design toward different regions of the design space, yielding markedly different link length configurations. This inherent tension precludes the existence of a single design that simultaneously achieves all individual optima, thereby motivating a systematic multi-objective optimization approach. In contrast to conventional weighted-sum aggregation, which requires a priori specification of weighting coefficients and may obscure non-convex regions of the Pareto front, this work adopts a Pareto-based multi-objective optimization framework that proceeds in three stages.

First, each objective $f_{k}$ (k=1,2,3) is maximized independently via full grid search over the discretized design space to identify its discrete numerical optimum, associated optimal length ratios, and sensitivity to dimensional variations (Section 3.1, Section 3.2 and Section 3.3). Second, all three objectives are evaluated at every feasible design point; the set of non-dominated solutions is extracted using the standard Pareto dominance criterion, and the conflict structure is quantified through Pearson correlation coefficients and the relative span of each objective on the Pareto front (Section 2.5 and Section 3.4). Third, guided by task priorities, a primary objective is first guaranteed at a high performance level, and within the resulting candidate subset, the remaining objectives are optimized via a normalized sum to yield a single recommended design that balances all three criteria (Section 3.5). This framework derives the final compromise directly from the objective trade-off landscape, avoiding the need for arbitrary pre-defined weights. The detailed mathematical formulations of $f_{1}$, $f_{2}$, and $f_{3}$ are provided in Section 2.2, Section 2.3 and Section 2.4, the Pareto analysis methodology is formalized in Section 2.5, and the complete optimization results are presented and discussed in Section 3.

### 2.2. Workspace Region-Based Optimization of Grasp Capability

#### 2.2.1. Grasping Mode Classification and Workspace Division

**Figure 2:**
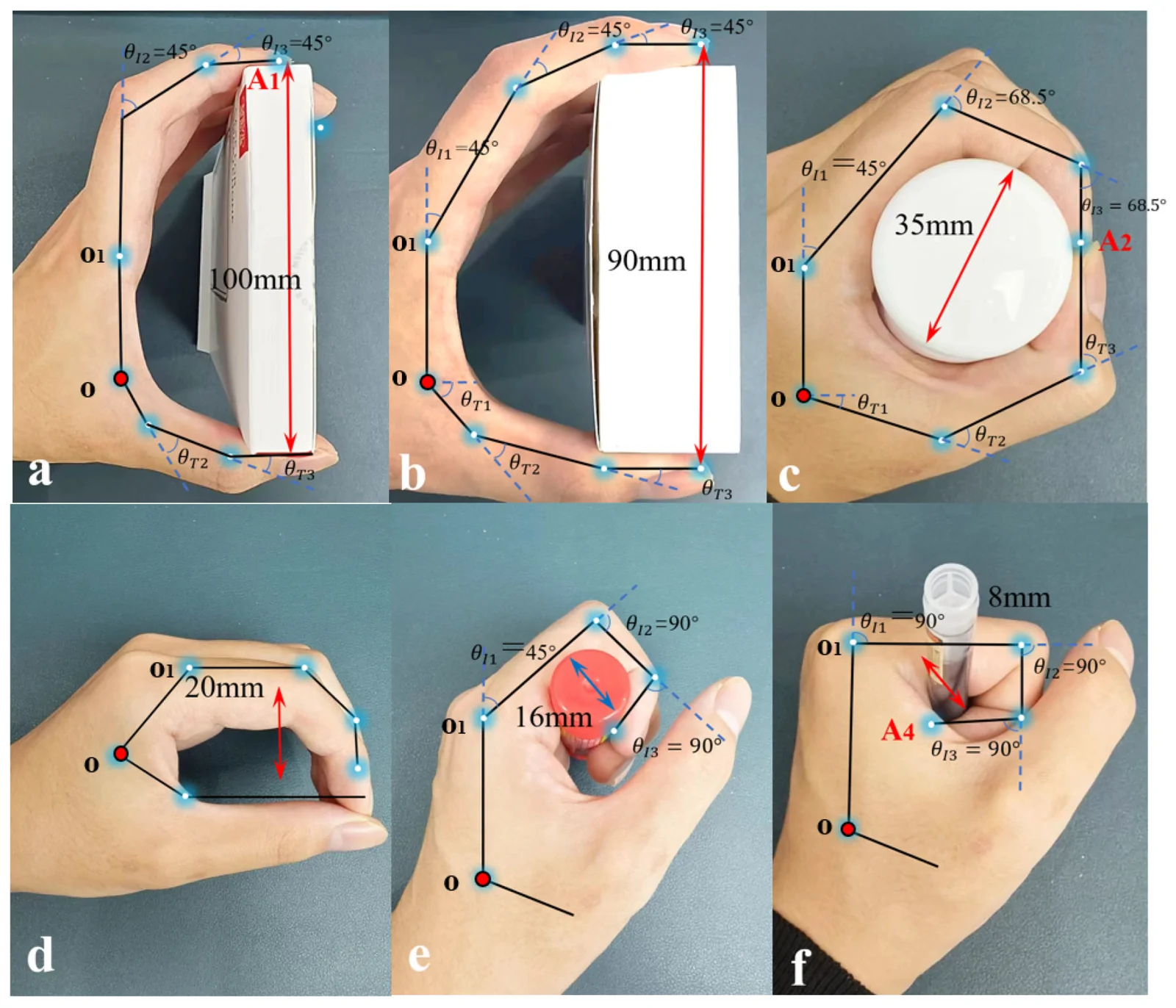
Schematic of grasping modes. (**a**–**d**) Double-enveloping grasp with successively decreasing enveloping range, providing stable force closure; (**e**,**f**) single-enveloping grasp, thumb not involved, compact structure adapted to narrow spaces.

To achieve force closure, the robotic hand primarily adopts the power grasp mode. Power grasping is further categorized into double-enveloping grasp and single-enveloping grasp. In double-enveloping grasping, the thumb and the index finger (together with other fingers) jointly form an envelope around the object. Figure 2a–d sequentially illustrate typical configurations with progressively reduced enveloping ranges. This grasping strategy provides a large contact area and stable force closure, making it suitable for gripping large or heavy objects. In single-enveloping grasping, only the index finger (or other fingers) and the palm form the envelope, while the thumb does not participate, as shown in Figure 2e,f. This configuration is more compact and well-suited for rapid grasping within confined spaces.

Workspace geometry, manipulability, and grasp-quality measures have been widely used to characterize kinematic reachability, grasp feasibility, and robustness [17,18,21,22,23,30]. Hand-posture subspace methods have also been used to represent and synthesize feasible grasp configurations [38,39]. These studies motivate the use of workspace geometry as an early-stage design proxy, although they do not establish a direct one-to-one relationship between workspace area and grasp success. These findings suggest that a geometrically defined workspace partition can serve as an effective proxy for evaluating grasp and manipulation performance. Motivated by this, the present work proposes a novel metric based on the area of a specific sub-region within the fingertip workspace, which captures the trade-off between power grasping and precision manipulation without requiring detailed object geometry or contact models.

**Figure 3:**
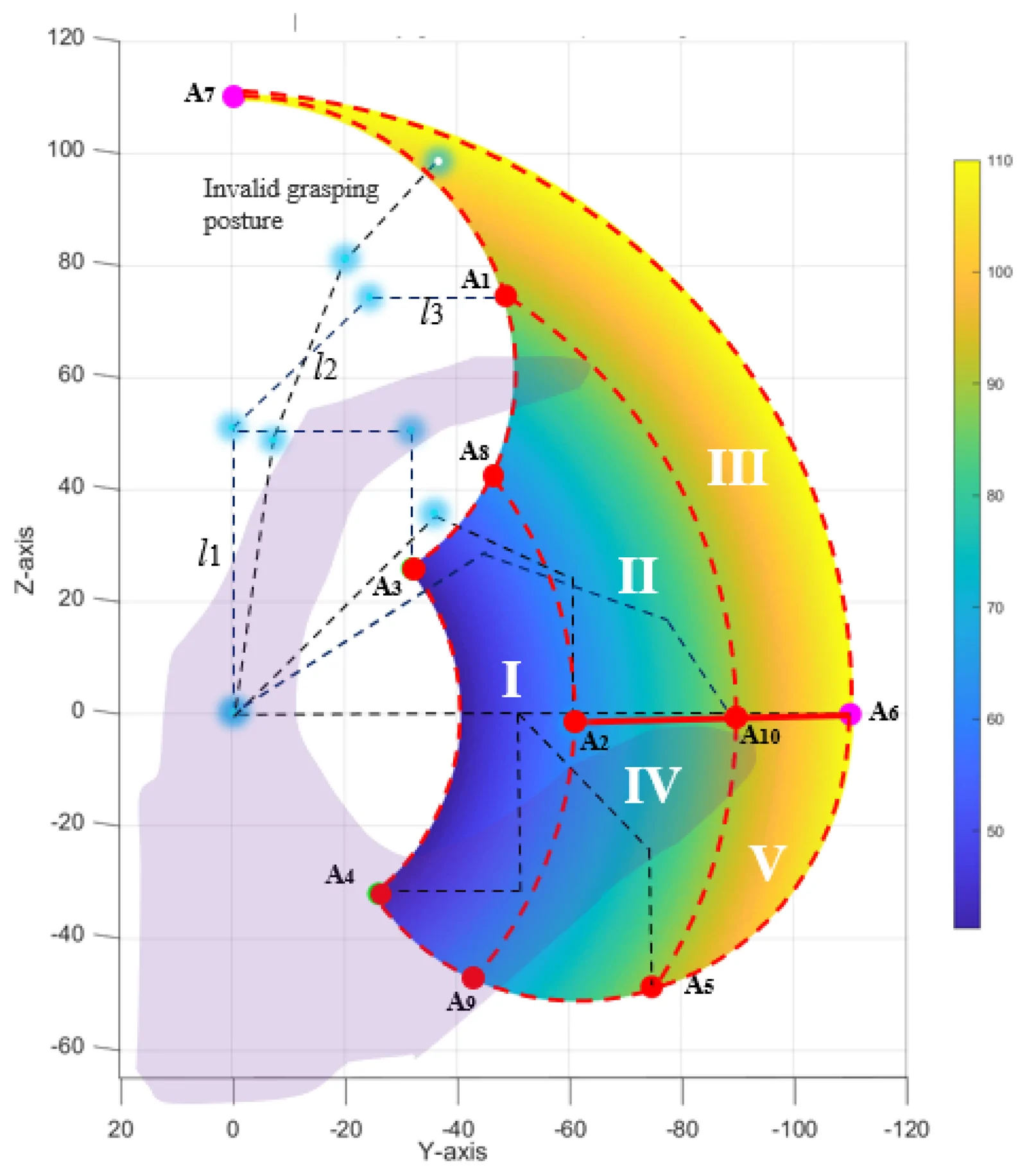
Workspace analysis diagram. Based on the three-rigid-link model and the coupling assumption $\theta_{3}=\theta_{4}$, characteristic posture points $A_{1}$–$A_{10}$ are marked, and the workspace is partitioned into five regions I–V.

Taking the index finger as the research object, several characteristic posture points are defined in Figure 3. Among them, $A_{1}$ corresponds to the upper-limit power-grasp posture ($\theta_{2}=0^{\circ }$, $\theta_{3}=\theta_{4}=45^{\circ }$, where $l_{1}$ remains vertical and $l_{3}$ remains horizontal); $A_{2}$ is the lower-limit posture (with $l_{3}$ kept vertical); $A_{3}$ corresponds to $\theta_{2}=0^{\circ }$, $\theta_{3}=\theta_{4}=90^{\circ }$, i.e., $l_{1}$ vertical, $l_{2}$ horizontal, $l_{3}$ vertical; $A_{4}$ corresponds to $\theta_{2}=90^{\circ }$, $\theta_{3}=\theta_{4}=90^{\circ }$, i.e., $l_{1}$ horizontal, $l_{2}$ vertical, $l_{3}$ horizontal; the curve $A_{3}A_{4}$ is the spatial trajectory formed by keeping $\theta_{3}=\theta_{4}=90^{\circ }$ constant while $\theta_{2}$ increases continuously from $0^{\circ }$ to $90^{\circ }$. Point $A_{5}$ ($\theta_{2}=90^{\circ }$, $\theta_{3}=\theta_{4}=45^{\circ }$, $l_{1}$ horizontal, $l_{3}$ vertical), $A_{6}$ ($\theta_{2}=90^{\circ }$, $\theta_{3}=\theta_{4}=0^{\circ }$, all three links horizontal), and $A_{7}$ ($\theta_{2}=0^{\circ }$, $\theta_{3}=\theta_{4}=0^{\circ }$, all three links vertical) are also clearly marked in the Figure 3. Curve $A_{3}A_{7}$ depicts the fingertip path when $\theta_{2}=0^{\circ }$ and $\theta_{3}=\theta_{4}$ increases uniformly from $0^{\circ }$ to $90^{\circ }$; curve $A_{7}A_{6}$ corresponds to the trajectory when $\theta_{3}=\theta_{4}=0^{\circ }$ and $\theta_{2}$ increases from $0^{\circ }$ to $90^{\circ }$; curve $A_{6}A_{4}$ corresponds to the trajectory when $\theta_{2}=90^{\circ }$ and $\theta_{3}=\theta_{4}$ increases from $0^{\circ }$ to $90^{\circ }$. Additionally, point $A_{8}$ is taken at $\theta_{2}=0^{\circ }$, $\theta_{3}=\theta_{4}=67.5^{\circ }$, point $A_{9}$ at $\theta_{2}=90^{\circ }$, $\theta_{3}=\theta_{4}=67.5^{\circ }$, $A_{10}$ is the fingertip position when $\theta_{2}=90^{\circ }$, $\theta_{3}=\theta_{4}=0^{\circ }$; and $A_{11}$ is defined as the intersection of the circle with center O and radius $OA_{8}$ and the positive x-axis, serving as an auxiliary point for area computation.

Based on the distribution of these characteristic points within the planar workspace, Figure 3. divides the workspace into five distinct sub-regions. Region I (bounded by $A_{3},A_{8},A_{9},A_{4}$) is surrounded by the trajectory of maximal flexion, representing deep power-grasp postures. Region II (bounded by $A_{1},A_{10},A_{2},A_{8}$) is the geometric overlap between the posture region assigned to power grasping and the posture region assigned to precision manipulation under the classification adopted in this study, thereby representing a kinematic grasp–manipulation compromise. Region III (bounded by $A_{7},A_{6},A_{10},A_{1}$) and Region V (bounded by $A_{10},A_{6},A_{5}$) both correspond to fully extended configurations. Region IV (bounded by $A_{2},A_{10},A_{5},A_{9}$) lies in an intermediate zone, bridging the flexed and extended postures. From the perspective of grasping tasks, power grasping typically requires the fingertip to enter a relatively large enveloping region; therefore, the fingertip positions are predominantly located in Regions I, II, and IV, which correspond to configurations with greater finger flexion and a fingertip located closer to the palm, and which are more conducive to achieving force and form closure. In contrast, precision manipulation demands high dexterity and positional resolution of the fingertip, often requiring the finger to be in a relatively extended yet precisely controllable state; thus, the fingertip is mainly located in Regions II and III, which better exploit the fingertip for contact tasks. Notably, under the geometric classification adopted in this study, Region II is the only region associated with both power-grasp and precision-manipulation configurations. Accordingly, its area is adopted as a kinematic proxy for evaluating the grasp–manipulation compromise, rather than as a direct measure of the overall capability of the robotic hand.

#### 2.2.2. Definition of Objective Function $f_{1}$

Unlike traditional grasp quality evaluation methods, such as force-closure margin or minimum grasping force, which typically require precise contact models and object geometry information, leading to complex computation and strong task dependence [19,20,21,22,23,39], the proposed Region II area metric is entirely based on the geometric division of the finger’s workspace. It requires neither a priori assumptions about object shape nor predefined contact point locations; instead, it directly quantifies the finger’s ability to compromise between power grasping and precision manipulation from a purely kinematic level. Compared with previous studies [28,29,30,40] that focus only on a single grasp mode or on the overall fingertip workspace envelope, the present work partitions the workspace into five functional regions and identifies the unique overlapping region (Region II) as a comprehensive performance index. This method provides a task-independent, a priori foundation for performance evaluation in the design of robotic hands.

Region II corresponds to the set of postures in which the finger transitions from a power grasp toward precision manipulation. Within this region, the finger can provide sufficient enveloping space for force closure while guaranteeing high fingertip positioning accuracy. The boundary of Region II consists of three geometric trajectories: curve $A_{1}A_{8}$ corresponds to the fingertip trajectory with $\theta_{2}=0^{\circ }$ and $\theta_{3}=\theta_{4}$ varying continuously from $45^{\circ }$ to $67.5^{\circ }$; curve $A_{8}A_{2}$ corresponds to the trajectory with $\theta_{3}=\theta_{4}=67.5^{\circ }$ and $\theta_{2}$ increasing from $0^{\circ }$ to $45^{\circ }$; curve $A_{10}A_{1}$ is the trajectory segment under the constraint $\theta_{3}=\theta_{4}=45^{\circ }$ with $\theta_{2}\in [0,arctan\;\frac{l_{2}sin\;45^{\circ }+l_{1}}{l_{2}cos\;45^{\circ }+l_{3}}]$. The coordinates of the characteristic points are as follows:

$$A_{1}=(l_{2}cos\;45^{\circ }+l_{3},l_{2}sin\;45^{\circ }+l_{1})$$

$$A_{2}=\left(\frac{sin\;67.5^{\circ }cos\;45^{\circ }}{\sin45^{\circ }}\;l_{2}+l_{1}cos\;45^{\circ },\frac{(cos\;45^{\circ }-1)sin\;67.5^{\circ }}{\sin45^{\circ }}\;l_{2}+l_{1}cos\;45^{\circ }-l_{3}\right)$$

$$A_{8}=(l_{2}sin\;67.5^{\circ }+l_{3}cos\;45^{\circ },l_{1}+l_{2}cos\;67.5^{\circ }-l_{3}sin\;45^{\circ })$$

$$A_{10}=\left(\sqrt{{\left(l_{2}sin\;45^{\circ }+l_{1}{)}^{2}+(l_{2}cos\;45^{\circ }+l_{3}\right)}^{2}},0\right)$$

$$A_{11}=\left(\sqrt{{\left(l_{2}sin\;67.5^{\circ }+l_{3}cos\;45^{\circ }{)}^{2}+(l_{1}+l_{2}cos\;67.5^{\circ }-l_{3}sin\;45^{\circ }\right)}^{2}},0\right)$$

**Figure 4:**
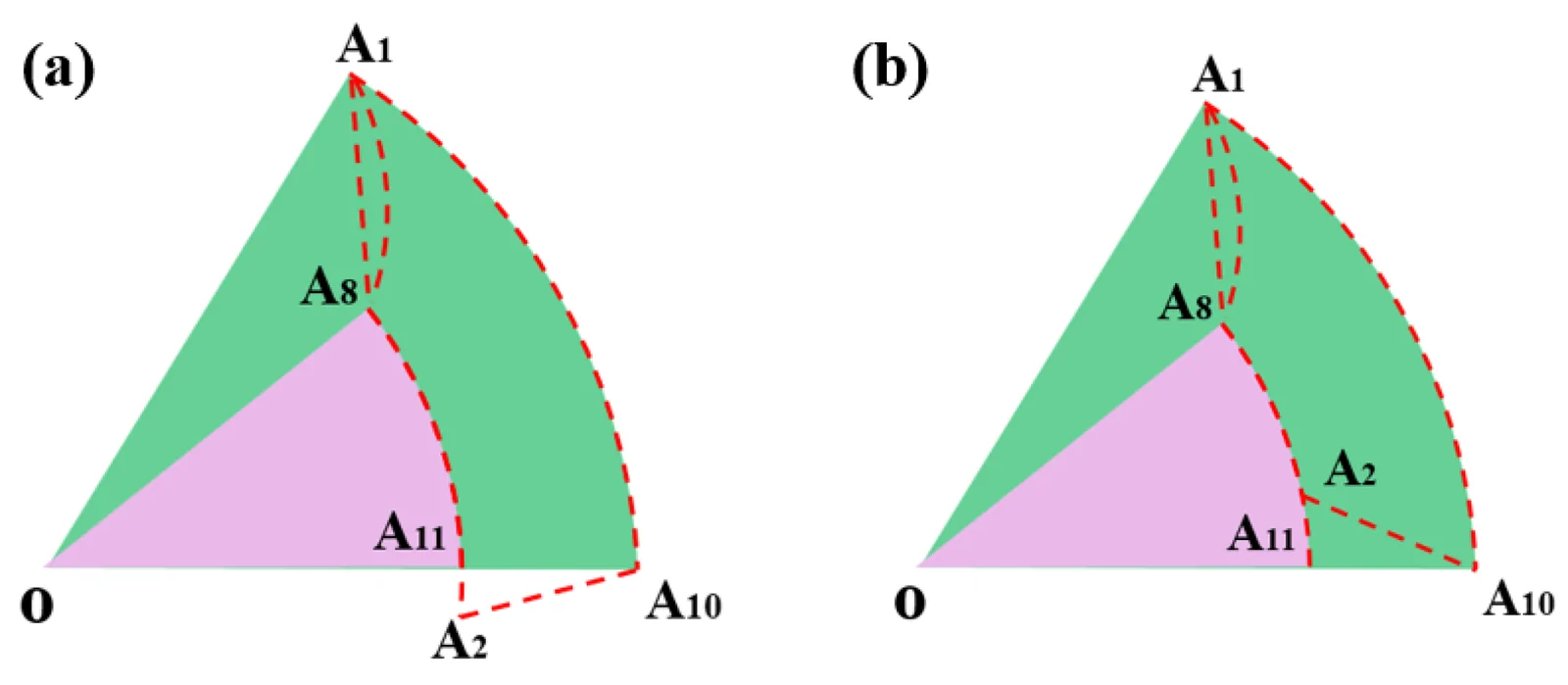
Schematic of the optimization calculation for Region II.

As illustrated in Figure 4, for convenient analytical computation, the area of Region II can be obtained through geometric decomposition: first calculate the area $S_{1}$ of sector $OA_{1}A_{10}$, subtract the area $S_{2}$ of sector $OA_{8}A_{11}$, then subtract the area $S_{3}$ of triangle $OA_{1}A_{8}$. $S_{4}$ is defined as the area of triangle $A_{2}A_{10}A_{11}$. When the y-coordinate of $A_{2}$ is greater than zero, $S_{4}$ is subtracted; when it is less than zero, $S_{4}$ is added. The objective function can therefore be written as

(1) $$f_{1}=S_{1}-S_{2}-S_{3}\pm S4,$$

where the sign of the last term follows the sign of $y_{A_{2}}$. All the above area components can be accurately computed from the three phalanx lengths $l_{1},l_{2},l_{3}$ and the joint angles corresponding to each characteristic point using planar geometry formulas. The link lengths satisfy the same total length and proportion constraints as defined in Section 2.1.

For a given pair $\left(l_{1},l_{3}\right)$, the value of $f_{1}$ is uniquely determined by the above formulas. A full grid search was performed over the constrained design space to identify the discrete numerical optimum. The design variables were uniformly sampled under the predefined length constraints, and the maximum $f_{1}$ together with the corresponding optimal lengths was selected. Specifically, letting O be the origin of the coordinate system, one has

(2) $$S_{1}=\frac{1}{2}|OA_{1}{|}^{2}\cdot ∠A_{1}OA_{10},$$

(3) $$S_{2}=\frac{1}{2}|OA_{8}{|}^{2}\cdot ∠A_{8}OA_{11},$$

(4) $$S_{3}=\frac{1}{2}|OA_{1}\times OA_{8}|,$$

(5) $$S_{4}=\frac{1}{2}|(A_{10}-A_{2})\times (A_{11}-A_{2})|,$$

A larger value of the objective function $f_{1}$ indicates a larger area of Region II, signifying a stronger overall capability of the robotic hand in both power grasping and precision manipulation. A larger Region II area indicates a broader set of kinematically feasible postures associated with both power-grasp and precision-manipulation configurations. It therefore provides more candidate configurations for subsequent grasp and manipulation planning. However, the proposed metric should be interpreted as an early-stage geometric design proxy rather than a direct measure of force closure, contact stability, or grasp robustness, because contact locations, friction constraints, and contact-force distributions are not included in the present model.

### 2.3. Kinematic Dexterity Optimization Based on the Condition Number

The condition number is a key metric for evaluating the kinematic performance of a manipulator. It reflects the uniformity of force and motion transmission in all directions at the end-effector [15,16,17,24,25] and is closely related to the system’s ability to avoid singular configurations. To improve the dexterous manipulation capability of the robotic hand, the condition number is taken as the second optimization objective in this work.

As shown in Figure 1, a simplified kinematic coordinate system is established for the dexterous hand. The end-effector coordinates $\left(x,y,z\right)$ are then expressed as:

(6) $$\begin{array}{cc} x & =[l_{3}cos\;(\theta_{2}+2\theta_{3})+l_{2}cos\;(\theta_{2}+\theta_{3})+l_{1}cos\;\theta_{2}]cos\;\theta_{1},\\ y & =[l_{3}cos\;(\theta_{2}+2\theta_{3})+l_{2}cos\;(\theta_{2}+\theta_{3})+l_{1}cos\;\theta_{2}]sin\;\theta_{1},\\ z & =l_{3}sin\;(\theta_{2}+2\theta_{3})+l_{2}sin\;(\theta_{2}+\theta_{3})+l_{1}sin\;\theta_{2}. \end{array}$$

Because the performance of a robotic hand should be evaluated over the entire workspace rather than at a single configuration, the objective function $f_{2}$ is defined as the global average of $\kappa^{-1}$ over a representative set of postures. Here, $\kappa (J_{\mathrm{eff}})$ denotes the condition number of the effective Jacobian matrix $J_{\mathrm{eff}}$, which maps joint velocities to end-effector velocities. Uniform sampling is employed: $\theta_{1}\in [-30^{\circ },30^{\circ }]$, $\theta_{2}\in [0^{\circ },90^{\circ }]$, $\theta_{3}\in [0^{\circ },90^{\circ }]$ with 10 nodes each, giving 1000 configurations. Thus,

(7) $$f_{2}\left(l_{1},l_{2},l_{3}\right)=\frac{1}{1000}\sum_{i=1}^{1000}\frac{1}{\kappa \left(J_{\mathrm{eff}}\left(\theta^{\left(i\right)}\right)\right)},\;$$

where $\theta^{\left(i\right)}=(\theta_{1},\theta_{2},\theta_{3}{)}_{i}$. A larger $f_{2}$ implies better global isotropy and greater average margin from singular configurations.

The design space and total-length constraint are as defined in Section 2.1. To obtain the numerical optimum within this discretized design space and to avoid convergence to local optima, a full grid search is performed instead of any gradient-based or stochastic method. The ranges of $l_{1}$ and $l_{2}$ are each discretized into 60 points, resulting in 3600 design parameter sets. For each set, $f_{2}$ is computed. The maximum obtained from the sampled design grid is reported as the discrete numerical optimum within the adopted discretized design space. The procedure is deterministic and repeatable. The optimization results, including the optimal link lengths and the corresponding maximum $f_{2}$ value, are presented in Section 3.2.

### 2.4. Key-Press Reachability Optimization

When a biomimetic robotic hand is employed for fine manipulation tasks such as keyboard typing, the range of keys that the fingertip can cover under a fixed wrist posture directly determines the operational efficiency and practicality. A larger static key-press span allows the hand to access multiple key rows without wrist motion, which enhances typing speed and reduces kinematic complexity. Ideally, the robotic hand should be capable of spanning multiple key rows rather than being confined to a single row. Therefore, the key-press range is adopted as the third optimization objective, denoted as $f_{3}$.

**Figure 5:**
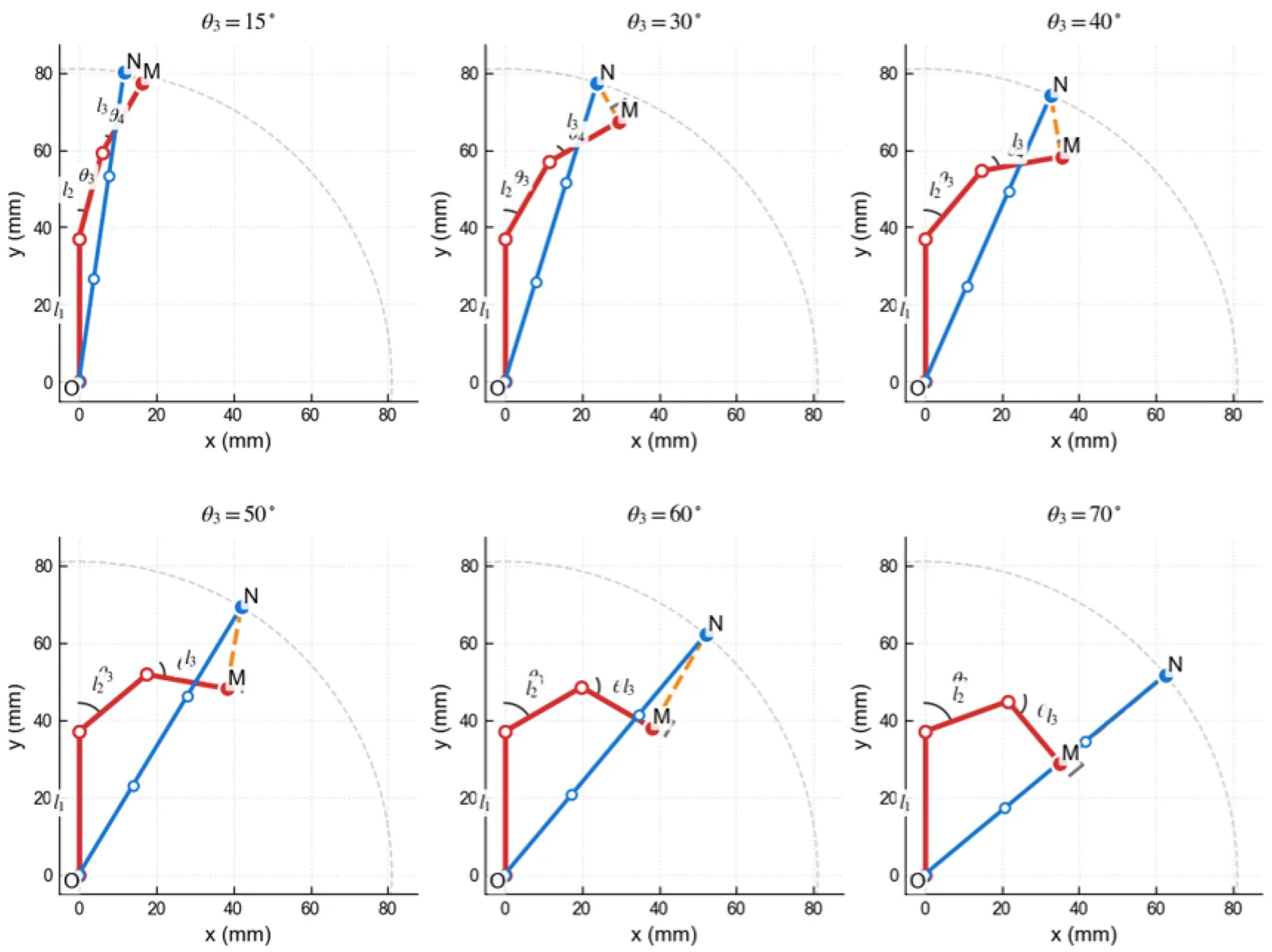
Finger postures at different $\theta_{3}$ ($15^{\circ }$–$70^{\circ }$) under the perpendicular constraint. Dashed orange lines mark the span MN. As $\theta_{3}$ increases, M moves downward/leftward while N slides along the maximum reach circle, showing how the perpendicular and slope conditions jointly define the feasible span.

As illustrated in Figure 5, two extreme fingertip positions on the key plane are defined: point M (the nearest point, corresponding to a fully flexed posture) and point N (the farthest point, corresponding to a fully extended posture). The length of segment MN thus represents the effective key-press range. Two practical kinematic constraints are imposed. First, to avoid jamming between keycaps, the distal phalanx must remain perpendicular to segment MN; any deviation from 90° may cause the fingertip to get stuck in the gaps during pressing. Second, the slope of the line from the wrist pivot point O (origin of the coordinate system) to point M must be smaller than the slope of MN; otherwise, the fingertip cannot geometrically reach the farthest point N (i.e., the finger would be blocked by its own base). Under these constraints, the optimization problem is formulated as maximizing MN, with the objective function denoted as $f_{3}=MN$.

Figure 5 further illustrates six typical postures with different values of the coupled joint angle $\theta_{3}$ (ranging from 15° to 70°). In each subfigure, the distal phalanx is kept strictly perpendicular to segment MN (marked by the right-angle symbol), and the corresponding MN span is indicated by a dashed orange line. It can be observed that as $\theta_{3}$ increases, point M moves downward and leftward, while point N slides along the maximum reach circle, resulting in a varying effective span. This series of plots demonstrates how the perpendicular constraint and the slope condition jointly determine the feasible MN for a given set of link lengths.

The link lengths satisfy the constraints specified in Section 2.1. For a given set of link lengths, the maximum feasible MN is determined by searching over the joint angle $\theta_{3}$ (with $\theta_{4}=\theta_{3}$) under the above constraints. The coordinates of points M and N are given by:

(8) $$\left\{\begin{array}{c} M_{x}=l_{2}sin\;\theta_{2}+l_{3}sin\;(\theta_{2}+\theta_{3}),\\ M_{y}=l_{1}+l_{2}cos\;\theta_{2}+l_{3}cos\;(\theta_{2}+\theta_{3}),\\ N_{x}=(l_{1}+l_{2}+l_{3})sin\;\theta_{1},\\ N_{y}=(l_{1}+l_{2}+l_{3})cos\;\theta_{1}, \end{array}\right.$$

where $\theta_{1}$ is the polar angle of point N on the maximum reach circle, and $\theta_{2}$ is derived from the perpendicular condition and the slope inequality.

To obtain the discrete numerical optimum, a full grid search was performed over the constrained design space. The variables $l_{1}$ and $l_{2}$ were uniformly sampled with 60 points each, while $l_{3}$ was calculated from the total-length constraint. The maximum feasible $f_{3}$ was then identified together with the corresponding optimal link lengths. The optimization results are presented in Section 3.3.

### 2.5. Multi-Objective Pareto Analysis

To systematically balance the three performance objectives, a Pareto-based multi-objective analysis is conducted [31,33]. All three objective functions are simultaneously evaluated over the design space defined in Section 2.1. A full grid search over the discretized design space was performed, where $l_{1}$ and $l_{2}$ were uniformly sampled with 60 points each, resulting in 3600 candidate design points. The corresponding $l_{3}$ values were calculated according to the fixed total-length constraint, and only feasible configurations satisfying all link length constraints were retained.

For a set of solutions $p_{i}=(f_{1}^{\left(i\right)},f_{2}^{\left(i\right)},f_{3}^{\left(i\right)})$, a solution $p_{i}$ is said to dominate $p_{j}$ (denoted $p_{i}≻p_{j}$) if and only if $f_{k}^{\left(i\right)}\geq f_{k}^{\left(j\right)}$ for all k∈{1,2,3} and at least one inequality is strict. The Pareto front consists of all non-dominated solutions. The 2-D Pareto fronts for each pair of objectives are extracted by projecting the three-dimensional solution set onto the corresponding planes. To quantify trade-offs, Pearson correlation coefficients between objectives are computed over all feasible points. The degree of conflict is further assessed by the relative span of each objective on the 3-D Pareto front compared to its full feasible range.

## 3. Results

### 3.1. Region II Optimization

**Figure 6:**
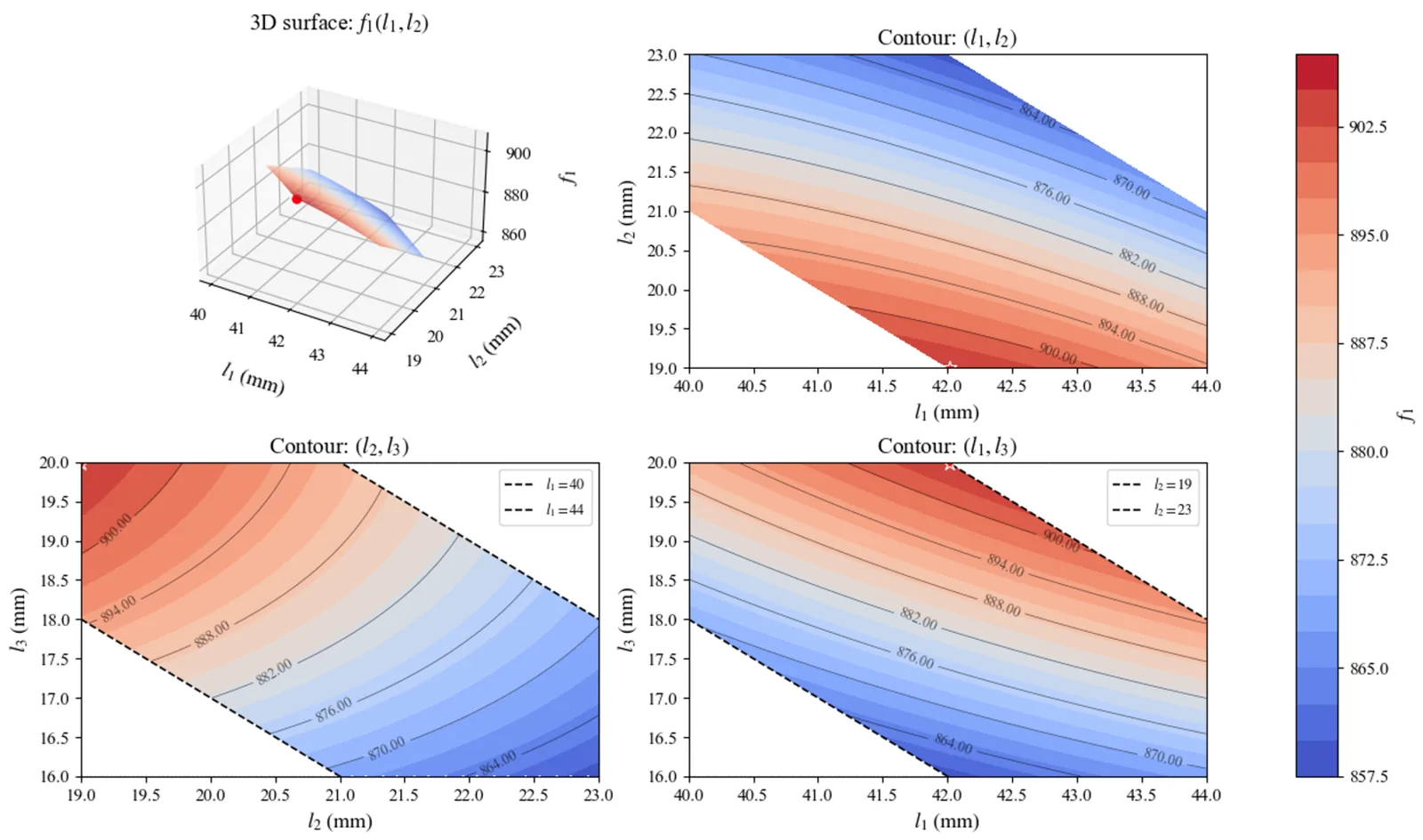
Optimization of $f_{1}$ under *κ* = 1.0. The four panels present the 3D surface and the contour projections in the $\left(l_{1},l_{2}\right)$, $\left(l_{2},l_{3}\right)$, and $\left(l_{1},l_{3}\right)$ planes. Red stars indicate the location of the maximum.

Following the workspace division and the definition of objective function $f_{1}$ established in Section 2.2, a full grid search was performed over the design space. The resulting $f_{1}$ surface and contour are shown in Figure 6.

The optimal combination is found at $l_{1}=42.03$ mm, $l_{2}=19.00$ mm, yielding $l_{3}=19.97$ mm and a maximum objective value of $f_{1}=905.19$ mm^2^. This indicates that to maximize the area of Region II (the transition region between power grasping and precision manipulation), the middle phalanx should be at its lower bound and the distal phalanx at its upper bound. As shown in Figure 6, $f_{1}$ increases as $l_{2}$ decreases and $l_{3}$ increases. The objective function varies smoothly, and the region near the optimum exhibits a gentle gradient, suggesting that small manufacturing deviations from these optimal lengths will not significantly degrade the grasp–manipulation compromise capability. The surface plot further confirms that the maximum is well-defined and lies on the boundary of the feasible domain.

These results provide a quantitative guideline for maximizing the transitional area: under the constant total-length constraint, a shorter middle phalanx combined with a longer distal phalanx is beneficial, while the proximal phalanx can be set near its upper bound without critically affecting the performance.

### 3.2. Condition Number Optimization Results

**Figure 7:**
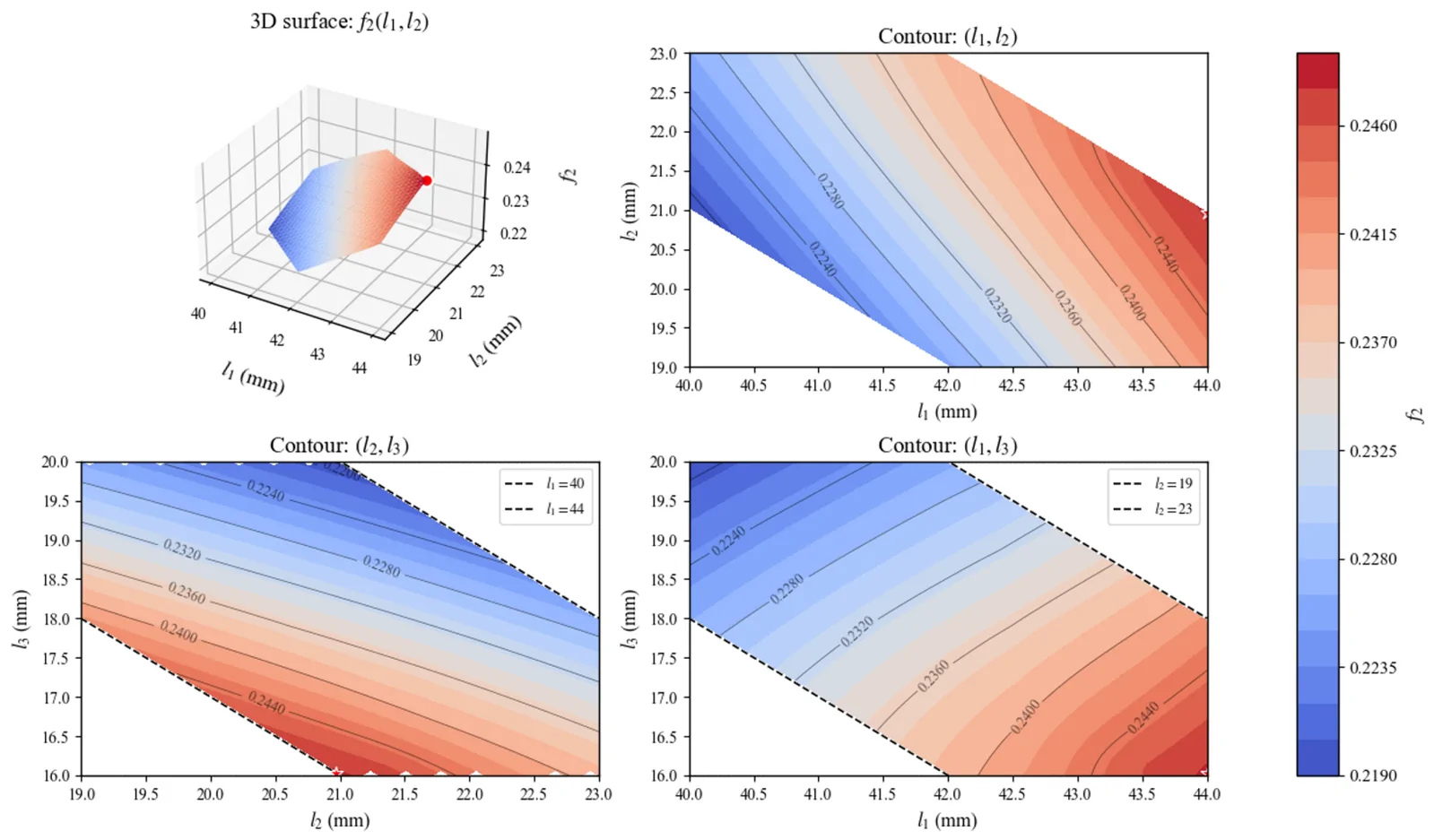
Optimization of $f_{2}$ under *κ* = 1.0. The four panels show the 3D surface and the contour projections in the $\left(l_{1},l_{2}\right)$, $\left(l_{2},l_{3}\right)$, and $\left(l_{1},l_{3}\right)$ planes. Red stars mark the maximum.

Following the method described in Section 2.3, the global dexterity index $f_{2}$ was optimized over the design space, and the results are shown in Figure 7. The optimal link lengths are found at $l_{1}=44.00$ mm, $l_{2}=20.97$ mm, yielding $l_{3}=16.03$ mm, with a corresponding maximum $f_{2}$ value of 0.2477. This indicates that to maximize the average reciprocal condition number over the 1000 sampled postures, the proximal phalanx should be as long as possible (upper bound), the distal phalanx as short as possible (lower bound), and the middle phalanx takes an intermediate value.

As shown in Figure 7, the 3D surface plot reveals a clear ridge of high dexterity along the direction of increasing $l_{1}$ and decreasing $l_{3}$. The contour projections consistently confirm that $f_{2}$ increases monotonically toward larger $l_{1}$ and smaller $l_{3}$, with the discrete numerical optimum located exactly on the boundary of the feasible domain.

The discretized grid search provides a numerical approximation of the optimum within the predefined design space. The smooth variation in *f*_2_ over the design space (as shown in Figure 7) confirms that no pathological local optima exist, and the maximum is well-defined on the boundary.

These results provide a quantitative guideline for improving kinematic dexterity: under the constant total-length constraint, a longer proximal phalanx combined with a shorter distal phalanx yields superior global isotropy and better singularity avoidance across the finger’s workspace.

### 3.3. Key-Press Range Optimization Results

**Figure 8:**
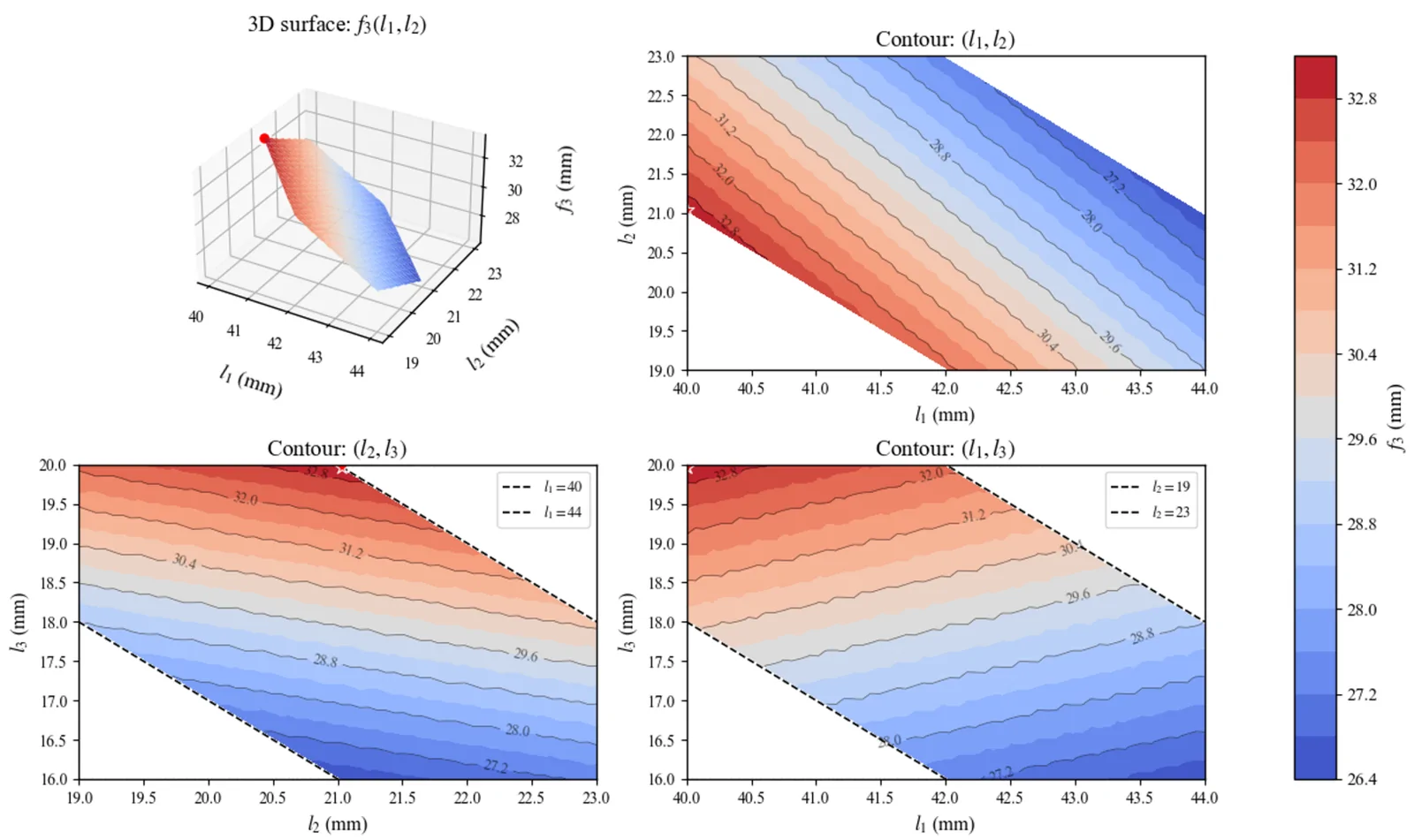
Optimization of $f_{3}$ under *κ* = 1.0. The four panels present the 3D surface and the contour projections in the $\left(l_{1},l_{2}\right)$, $\left(l_{2},l_{3}\right)$, and $\left(l_{1},l_{3}\right)$ planes. Red stars mark the maximum.

Following the method described in Section 2.4, the key-press range $f_{3}$ was maximized over the same design space and under the identical total-length constraint. The resulting surface and contour plots are shown in Figure 8.

The optimal link lengths are found at $l_{1}=40.00$ mm, $l_{2}=21.03$ mm, yielding $l_{3}=19.97$ mm, and the corresponding maximum key-press span is $f_{3}=33.07$ mm. This indicates that to maximize the static reachability under the perpendicular and slope constraints, the proximal phalanx should be near its lower bound, the middle phalanx near its upper bound, and the distal phalanx near the upper bound of the feasible range.

As shown in Figure 8, the 3D surface plot and contour projections indicate that $f_{3}$ increases as $l_{1}$ decreases and $l_{3}$ increases, with the discrete numerical optimum located exactly on the boundary of the feasible domain.

These results provide a clear design guideline for improving fingertip reachability in fine manipulation tasks: a shorter proximal phalanx together with a longer distal phalanx maximizes the static key-press range, enabling the robotic hand to cover more key rows without wrist movement.

### 3.4. Multi-Objective Pareto Analysis Results

Over the entire feasible design space, the ranges of the three objectives are as follows: $f_{1}$ varies from 858.78 to 905.19 (a relative increase of about 5.4%), $f_{2}$ from 0.2192 to 0.2477 (about 13.0%), and $f_{3}$ from 26.53 mm to 33.07 mm (about 24.7%). The key-press span $f_{3}$ exhibits the largest variation, indicating that it is highly sensitive to the allocation of link lengths, whereas the area of Region II ($f_{1}$) remains relatively stable across the design space.

The Pearson correlation coefficients between the objectives reveal a strong negative correlation between $f_{2}$ and $f_{3}$ (r=−0.9550), and a moderate negative correlation between $f_{1}$ and $f_{2}$ (r=−0.4391), while $f_{1}$ and $f_{3}$ show a positive correlation (r=0.6824). This indicates a fundamental trade-off: designs that maximize the key-press range inevitably sacrifice kinematic dexterity, and designs that maximize Region II area also tend to reduce dexterity, but Region II area and key-press range are positively aligned.

**Figure 9:**
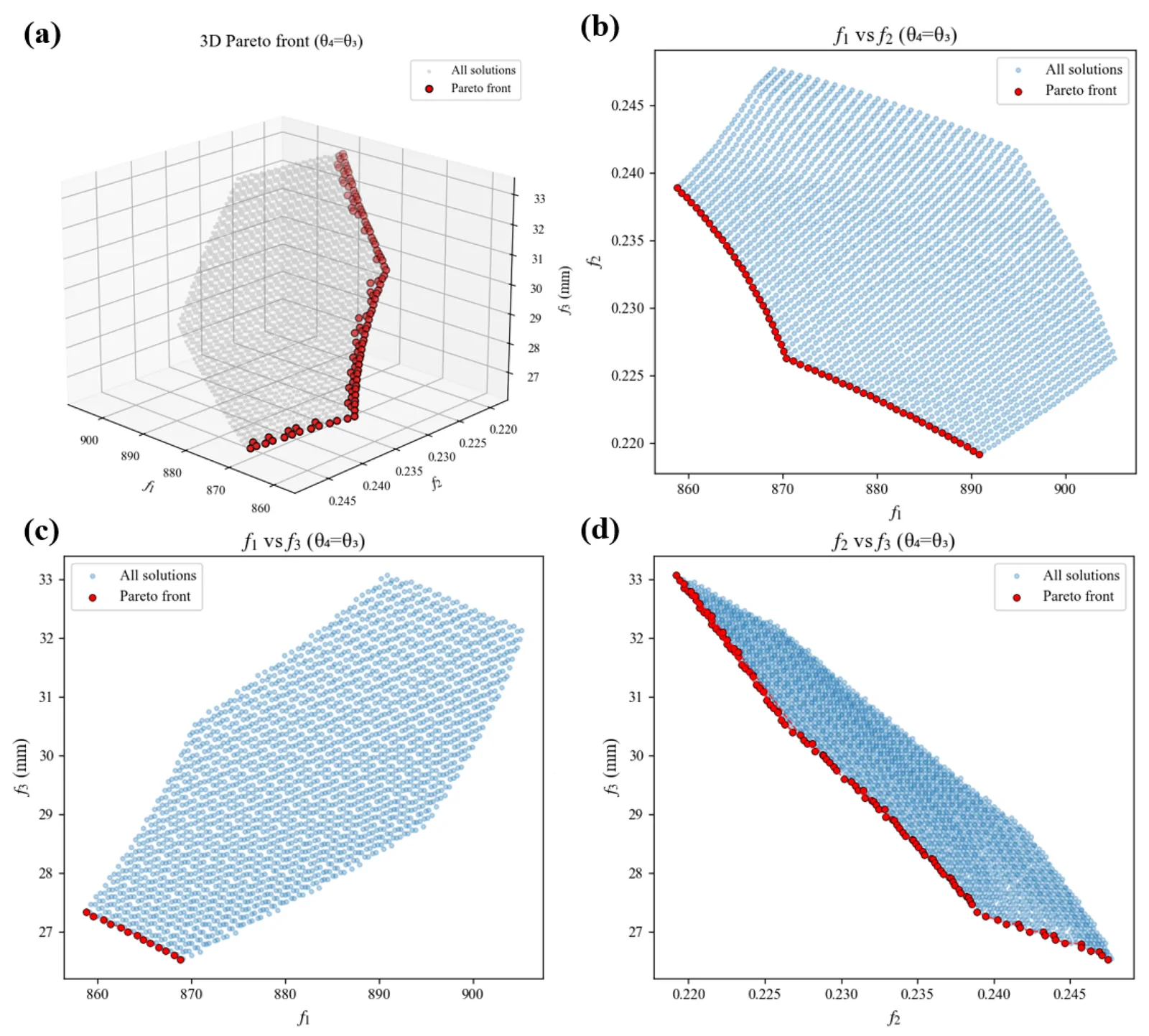
Pareto front analysis. (**a**) 3D scatter plot of all feasible (gray) and Pareto optimal (red) solutions. (**b**) $f_{1}$ vs. $f_{2}$; (**c**) $f_{1}$ vs. $f_{3}$; (**d**) $f_{2}$ vs. $f_{3}$. Red points and dashed lines denote the Pareto front.

As shown in Figure 9, the three-dimensional Pareto front contains 117 non-dominated points, and the relative spans of the objectives on the frontier are 70.2% for $f_{1}$, 99.2% for $f_{2}$, and 100% for $f_{3}$, confirming that the entire feasible ranges of dexterity and key-press span are in direct conflict, while $f_{1}$ covers most but not all of its full feasible range.

Because the key-press span is the most sensitive objective (24.7% variation) and is directly responsible for the robotic hand’s ability to perform fine manipulation tasks such as keyboard typing without wrist movement, it is chosen as the primary optimization criterion. A hierarchical selection strategy is therefore adopted: from the three-dimensional Pareto front, only solutions with $f_{3}$ at least 95% of its global maximum (i.e., $f_{3}\geq 31.42$ mm) are retained. This subset contains 19 candidate designs. Within this subset, $f_{1}$ and $f_{2}$ are normalized to the range $\left[0,1\right]$ using their global minima and maxima, and the solution that maximizes the sum of the normalized values is selected as the recommended compromise design. This approach prioritizes the most critical objective while maintaining reasonable performance in the other two. The recommended design is $l_{1}=40.44$ mm, $l_{2}=21.39$ mm, $l_{3}=19.17$ mm, yielding $f_{1}=894.42$, $f_{2}=0.2213$, and $f_{3}=32.40$ mm. This solution achieves 98.0% of the maximum possible key-press span, while preserving competitive $f_{2}$ performance without significant degradation relative to the extreme $f_{3}$-maximizing design, and maintaining $f_{1}$ within 1.2% of its maximum. Thus, it provides a well-balanced trade-off suitable for practical prototyping.

### 3.5. Experimental Validation

**Figure 10:**
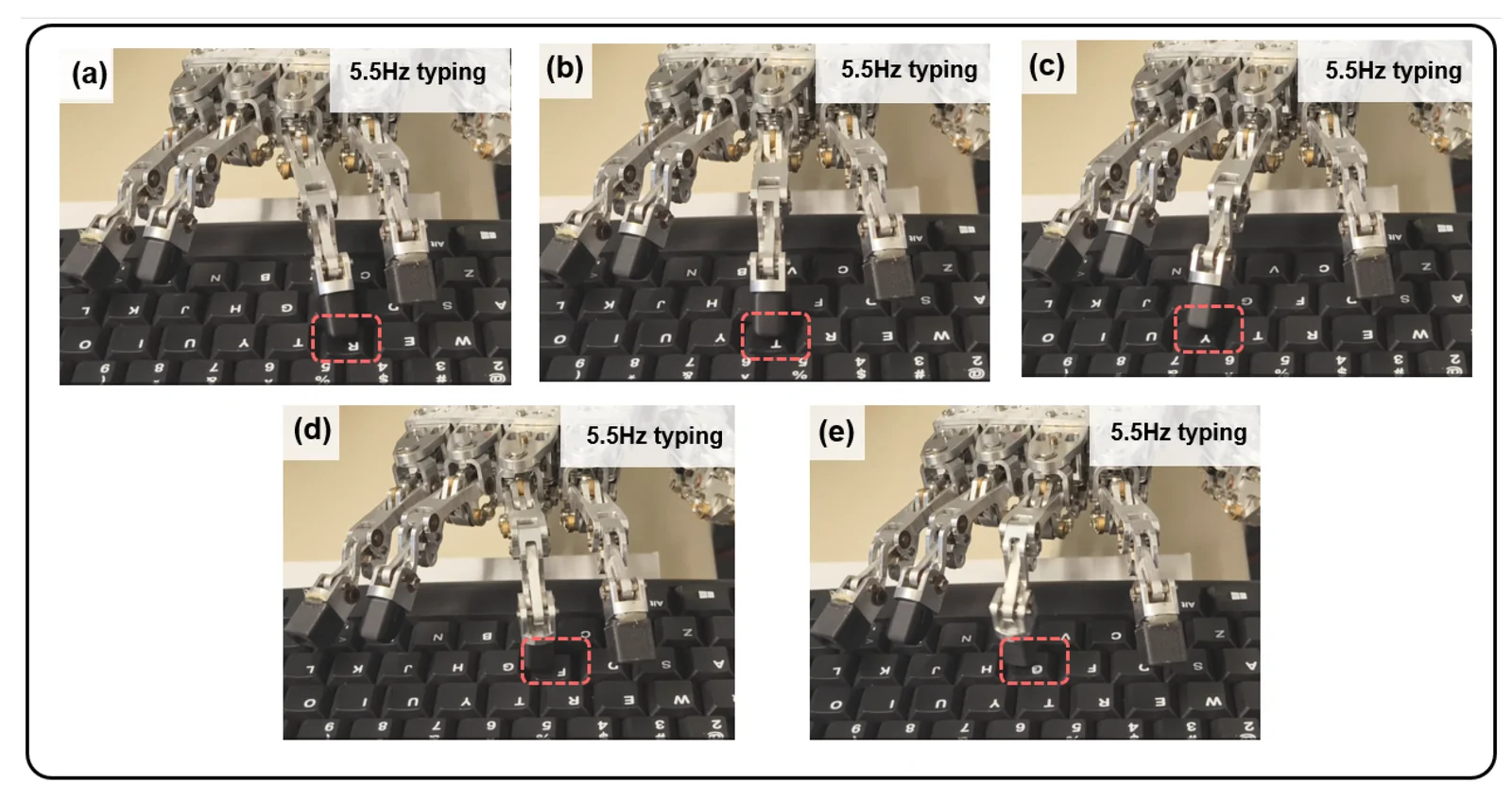
Experimental verification of the key-press range optimization.

Using the optimized dimensions obtained in Section 3.4 ($l_{1}=40.44\;\mathrm{mm}$, $l_{2}=21.39\;\mathrm{mm}$, $l_{3}=19.17\;\mathrm{mm}$), a robotic hand prototype was fabricated and mounted on a fixed-wrist platform for typing experiments. As shown in Figure 10a–e, the middle finger of the prototype easily achieved a stable keystroke frequency of approximately 5.5 Hz, reliably striking multiple keys across different rows—including “R”, “T”, “Y”, “F”,and “G”—without any wrist movement. The fingertip motion remained smooth throughout the entire process, and no jamming into the gaps between keycaps was observed.

The quantitative keystroke reliability evaluation results are summarized in Table 1. For each of the five tested keys, 200 striking trials were conducted, resulting in a total of 1000 trials. The success rate of each key exceeds 97.5%, and the overall success rate reaches 98.8%. The few unsuccessful triggers are mainly attributed to incomplete key travel caused by an excessively fast striking frequency.

### 3.6. Effect of Joint Coupling Coefficient on Optimal Design

To verify robustness to the coupling assumption, the analysis in Section 3.1, Section 3.2 and Section 3.3 was repeated under *κ* = 0.7 ($\theta_{4}$ = 0.7$\theta_{3}$) and compared against the *κ* = 1 baseline.

#### 3.6.1. Grasp-Capability Optimization Under *κ* = 0.7

**Figure 11:**
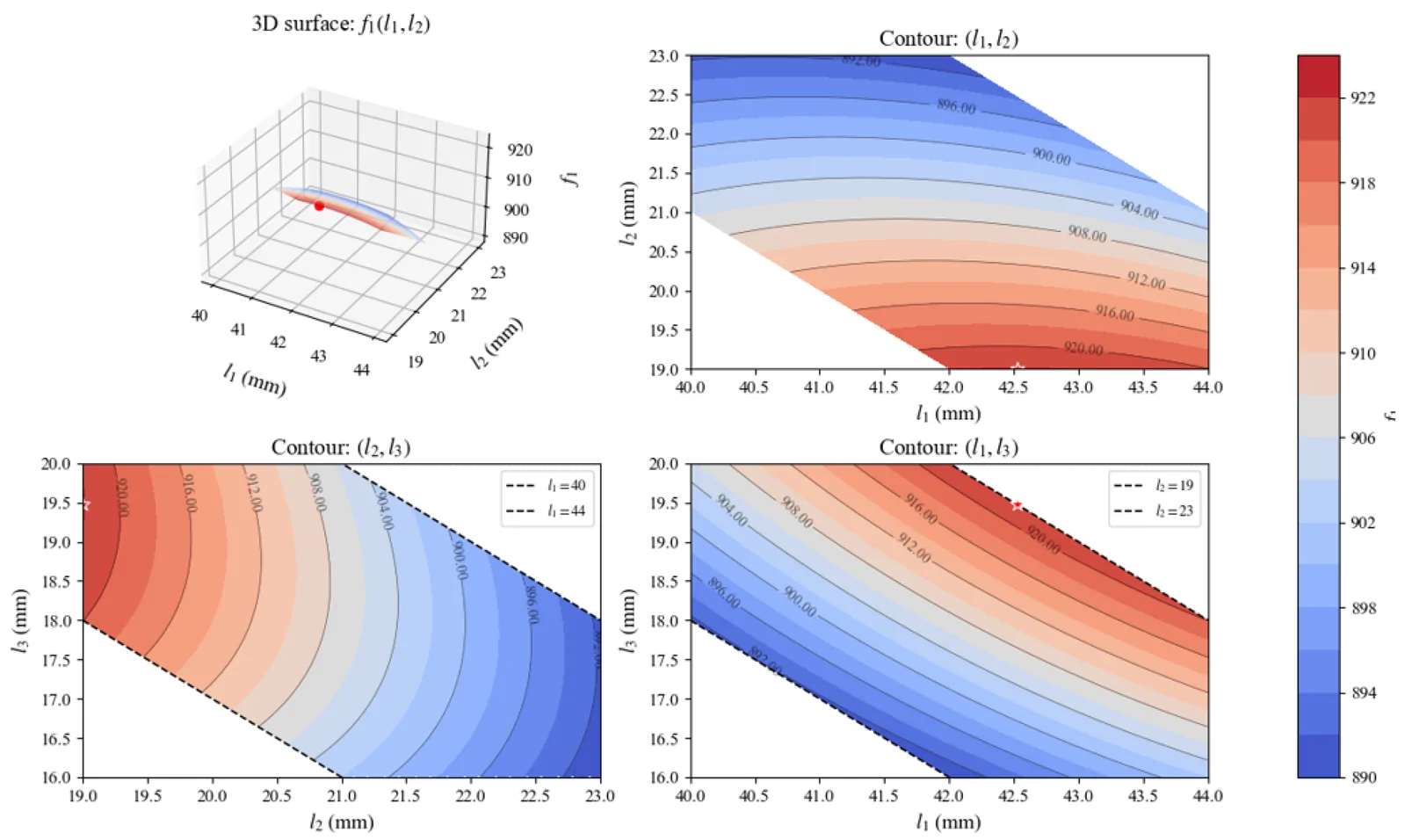
Optimization of $f_{1}$ under *κ* = 0.7. The four panels present the 3D surface and the contour projections in the $\left(l_{1},l_{2}\right)$, $\left(l_{2},l_{3}\right)$, and $\left(l_{1},l_{3}\right)$ planes. Red stars indicate the location of the maximum.

Following the same workspace division method established in Section 2.2, the Region II area was recomputed with the updated posture geometry. A full grid search over the identical design space was performed, and the resulting $f_{1}$ surface and contours are shown in Figure 11.

The optimal combination under *κ* = 0.7 is found at $l_{1}=42.53$ mm, $l_{2}=19.00$ mm, yielding $l_{3}=19.47$ mm and a maximum objective value of $f_{1}=922.10$ mm^2^. Similar to the *κ* = 1 case (Section 3.1), the maximum lies on the boundary of the feasible domain with $l_{2}$ at its lower bound, confirming that a shorter middle phalanx combined with a longer distal phalanx remains favorable for maximizing the transitional workspace area. The objective function $f_{1}$ increases as $l_{2}$ decreases and $l_{3}$ increases.

#### 3.6.2. Kinematic Dexterity Optimization Under *κ* = 0.7

**Figure 12:**
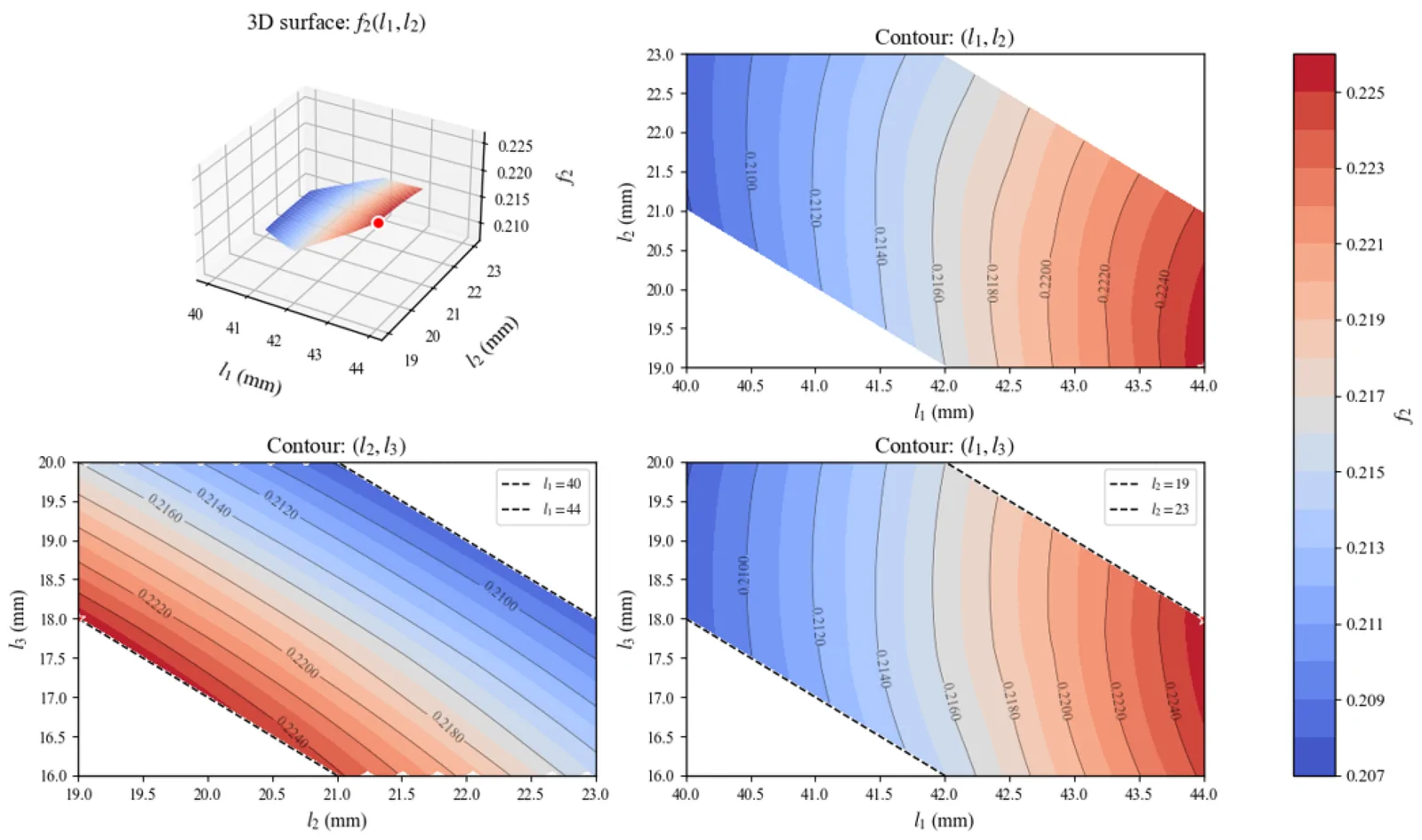
Optimization of $f_{2}$ under *κ* = 0.7. The four panels show the 3D surface and the contour projections in the $\left(l_{1},l_{2}\right)$, $\left(l_{2},l_{3}\right)$, and $\left(l_{1},l_{3}\right)$ planes. Red stars mark the maximum.

The global dexterity index $f_{2}$ was recomputed by averaging the reciprocal condition number over the joint space with the revised Jacobian that incorporates the 0.7 coupling coefficient. The optimization results are shown in Figure 12.

The optimal link lengths under *κ* = 0.7 are found at $l_{1}=44.00$ mm, $l_{2}=19.33$ mm, yielding $l_{3}=17.67$ mm, with a corresponding maximum $f_{2}$ value of 0.2281. Consistent with the *κ* = 1 result (Section 3.2), the proximal phalanx remains at its upper bound (*l*_1_ = 44 mm), as increasing *l*_1_ consistently enlarges the workspace radius R and improves the overall kinematic conditioning. However, when the coupling ratio decreases to *κ* = 0.7, the distal phalanx no longer benefits from being minimized. The reason lies in the structure of the Jacobian’s third column (corresponding to ∂/∂*θ*_3_), where *l*_3_ appears with an effective coefficient of (1 + *κ*). When *κ* = 1.0, this coefficient equals 2, making the third column norm excessively large; reducing *l*_3_ brings the column norms toward a balanced, isotropic configuration and thus increases *f*_2_. When *κ* = 0.7, the coefficient drops to 1.7, and the third column norm is already closer to the others. Further reducing *l*_3_ would instead drive the norm ratio *n*_1_/*n*_3_ away from unity and degrade dexterity. Consequently, the optimization shifts priority toward shortening the middle phalanx (*l*_2_ drops from 20.97 mm to 19.33 mm), which simultaneously reduces the *l*_2_-contributed components in both the second and third columns, yielding a more balanced set of column norms. The distal phalanx, in turn, adopts a longer value (*l*_3_ increases from 16.03 mm to 17.67 mm). Thus, while the qualitative guideline—maximizing the proximal phalanx—remains valid, the trade-off between the middle and distal phalanges is governed by the coupling-induced weighting (1 + *κ*) in the Jacobian, and the optimal *l*_3_ increases as κ decreases.

#### 3.6.3. Key-Press Range Optimization Under *κ* = 0.7

**Figure 13:**
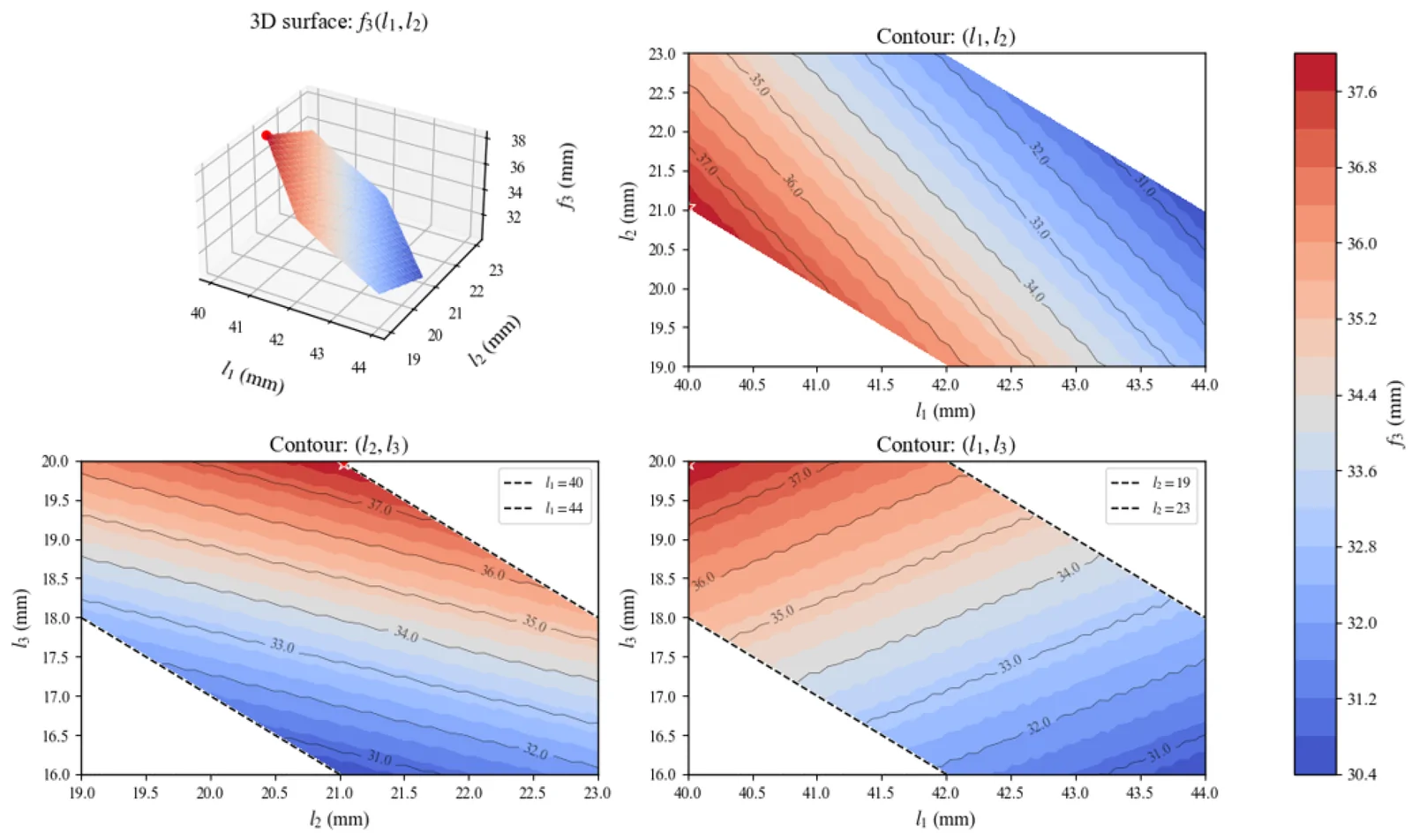
Optimization of $f_{3}$ under *κ* = 0.7. The four panels present the 3D surface and the contour projections in the $\left(l_{1},l_{2}\right)$, $\left(l_{2},l_{3}\right)$, and $\left(l_{1},l_{3}\right)$ planes. Red stars mark the maximum.

The key-press range optimization was repeated under the perpendicularity and slope constraints with the revised kinematics. The results are shown in Figure 13.

The optimal link lengths under *κ* = 0.7 are found at $l_{1}=40.10$ mm, $l_{2}=20.95$ mm, yielding $l_{3}=19.95$ mm, and the corresponding maximum key-press span is $f_{3}=37.86$ mm. Notably, the optimal length configuration is virtually identical to that under *κ* = 1, indicating that the key-press range is insensitive to the exact coupling ratio. The objective landscape continues to exhibit a strong ridge along the direction of decreasing $l_{1}$ and increasing $l_{3}$, confirming that a shorter proximal phalanx combined with a longer distal phalanx maximizes the static key-press span regardless of the coupling coefficient.

#### 3.6.4. Trend Consistency and Design Robustness

A quantitative comparison of the single-objective optima under *κ* = 1 and *κ* = 0.7 is summarized in Table 2. Although the absolute optimal values shift in response to the reduced coupling coefficient, the qualitative design guidelines derived in Section 3.1, Section 3.2 and Section 3.3 remain fully preserved:

For $f_{1}$, the optimum consistently lies at the lower bound of $l_{2}$, with $l_{3}$ taking a large value, indicating that a short middle phalanx benefits the transitional workspace area under both coupling assumptions.

For $f_{2}$, the optimum consistently pushes $l_{1}$ to its upper bound, while the behavior of $l_{3}$ depends on κ: at κ=1.0, $l_{3}$ approaches its lower bound (16.03 mm), confirming that a short distal phalanx improves global isotropy under full coupling; at κ=0.7, however, $l_{3}$ increases (to 17.67 mm), showing that the benefit of a short distal phalanx diminishes as the coupling ratio decreases.

For $f_{3}$, the optimum consistently favors a small $l_{1}$ and large $l_{3}$, indicating that fingertip reachability is maximized by allocating length to the distal end of the finger.

These results demonstrate that, although the optimal $l_{3}$ for $f_{2}$ varies with the coupling ratio (increasing from 16.03 mm to 17.67 mm as κ decreases from 1.0 to 0.7), the overall directional influences of each phalanx length on the three performance metrics are qualitatively robust. Consequently, the hierarchical selection strategy presented in Section 3.5—which prioritizes $f_{3}$ and then balances $f_{1}$ and $f_{2}$—remains applicable for designs employing alternative coupling ratios within the physiologically observed range.

## 4. Discussion

The most distinctive contribution of this work is the introduction of Region II as a workspace zone that belongs simultaneously to power grasp and precision manipulation postures. We further demonstrate that the area of this region can be tuned by redistributing phalanx lengths under a fixed total length. Unlike conventional grasp metrics that rely on contact models or object geometry, the area of Region II serves as a purely kinematic, task-agnostic proxy for the grasp-versus-manipulation trade-off. Parametric sweeps consistently show that a shorter middle phalanx, pushed to its lower bound, paired with a longer distal phalanx enlarges this shared region without increasing the overall finger size. This trend echoes the biomechanical proportions of the human hand, where the middle phalanx acts as a key lever for both powerful grips and fine motor actions. This suggests that the existence of a compromise workspace is rooted in fundamental kinematic geometry rather than implementation specifics.

On the use of Region II area as a grasp-capability indicator. We acknowledge that a complete assessment of grasp capability should also consider contact locations and force directions, as emphasized in classical grasp quality measures [19,20,21,22,23]. However, as reviewed in Section 2.2.1, existing studies have established positive correlations between workspace geometry and grasp performance: the size of high-manipulability regions correlates with grasp success rate [22,30], and the volume of the force-closure workspace reflects grasp tolerance to uncertainties [21,22,23]. Region II is thus not intended to replace those detailed contact-based metrics, but rather to provide a kinematic, a priori design proxy that captures the geometric compromise between power and precision grasps. It is most useful in early-stage dimensional synthesis where object geometry and contact points are not yet specified. By maximizing this shared workspace area, we increase the set of postures that can simultaneously meet the gross geometric requirements of both grasp modes, thereby enhancing the likelihood of achieving force closure and fine control without requiring detailed object models. Furthermore, the current two-dimensional planar analysis is directly applicable to objects with a dominant planar interaction (e.g., cylinders, prismatic parts, keyboard typing). For general three-dimensional objects, a full 3D workspace analysis would be necessary. We have therefore added this as a clear limitation and a direction for future work. The proposed grasp-capability metric $f_{1}$ is purely kinematic and does not account for contact force distribution, friction constraints, or dynamic effects. Future work will integrate force-closure criteria, such as minimum grasping force or force-closure margin, with the workspace-based metric to provide a more complete assessment of grasp stability, following established approaches in the literature.

We explicitly clarify why the three specific sub-objectives were chosen to realize a highly generalisable biomimetic finger. Each criterion addresses a distinct and indispensable facet of finger functionality. Grasp capability ($f_{1}$) captures the finger’s ability to stably prehend objects, covering both power grasping (force closure) and precision manipulation (controlled fingertip contact). Kinematic dexterity ($f_{2}$) quantifies the uniformity of motion and force transmission across the workspace via the condition number, reflecting how well the finger can avoid singular configurations and perform arbitrary fine motions. It is important to clarify that a condition number close to 1 primarily indicates isotropy (i.e., uniform manipulability in all directions) rather than directly measuring absolute positioning accuracy or the overall functional precision of the entire hand. Absolute positioning accuracy also depends on joint resolution, control bandwidth, structural stiffness, and sensor feedback, which are not captured by the kinematic condition number. Within our framework, $f_{2}$ serves strictly as a kinematic dexterity metric—one of three complementary objectives—and should not be overinterpreted as a comprehensive measure of hand-level functional precision. Key-press reachability ($f_{3}$) represents the finger’s ability to reach and interact with specific points in the workspace under realistic perpendicularity and slope constraints, which is essential for tasks such as typing, button pressing, or touchscreen operation. Together, these three criteria span the functional spectrum from coarse prehension ($f_{1}$) to motion quality ($f_{2}$) to targeted fine interaction ($f_{3}$). We acknowledge that they do not exhaustively cover all aspects of hand performance (e.g., dynamic force exertion, impact resilience, tactile sensing integration). Nevertheless, the proposed framework is intentionally modular and extensible, allowing additional objectives to be incorporated in future work.

When the three objectives are considered together, the most severe conflict emerges not between grasp capability and dexterity, but between dexterity ($f_{2}$) and key-press range ($f_{3}$), with a Pearson correlation of r=−0.955, a near-perfect negative relationship. In contrast, the correlation between grasp capability ($f_{1}$) and dexterity ($f_{2}$) is only moderate (r=−0.439), indicating a milder tension. Interestingly, $f_{1}$ and $f_{3}$ are positively correlated (r=0.682). Consequently, designs that extend the static key-press span do not necessarily compromise the grasp–manipulation compromise; they may even slightly improve it. Among the three metrics, the key-press range exhibits the highest sensitivity to dimensional changes, a 24.7% spread across the design space, whereas the Region II area is comparatively robust with only 5.4% variation. This high sensitivity makes $f_{3}$ the most discriminating design metric and therefore the most critical to validate experimentally. The three-dimensional Pareto front contains 117 non-dominated solutions. The relative spans of $f_{1}$, $f_{2}$, and $f_{3}$ on the frontier are 70.2%, 99.2%, and 100%, respectively. This confirms that the entire feasible ranges of dexterity and key-press range are in direct opposition, while grasp capability covers most, but not all, of its full feasible range.

Given that key-press range is the most sensitive and directly task-relevant for fine manipulation (e.g., typing without wrist motion), a hierarchical selection strategy is adopted. First, we retain designs that achieve at least 95% of the maximum $f_{3}$. Within that subset, we then maximize the normalized sum of $f_{1}$ and $f_{2}$. The recommended compromise design has $l_{1}=40.44$ mm, $l_{2}=21.39$ mm, $l_{3}=19.17$ mm. It reaches 98.0% of the peak key-press span, improves $f_{2}$ by 1.0% relative to the extreme $f_{3}$-maximizing design, and stays within 1.2% of the maximum $f_{1}$. A physical prototype built to these dimensions achieved a 98.8% success rate over 1000 cross-row strikes at approximately 5.5 Hz without wrist movement. This confirms that the perpendicularity and slope constraints effectively prevent jamming. It must be noted, however, that the experimental validation currently covers only $f_{3}$. The best $f_{1}$ and $f_{2}$ values were numerically identified within the adopted discretized design space, but their predicted performance advantages have not yet been physically validated. Furthermore, the typing experiment was conducted using only the recommended compromise phalanx lengths, at a single typing speed (approximately 5.5 Hz), and without direct comparison against other phalanx ratios (e.g., $f_{1}$-dominant, $f_{2}$-dominant, or uniform lengths) or existing robotic hands. These limitations mean that the relative performance advantages of the recommended design over alternative configurations or state-of-the-art hands have not been experimentally quantified. Future work should experimentally characterize fingers optimized for $f_{1}$-dominant or $f_{2}$-dominant configurations to close this gap, as well as perform comparative studies across different speeds, phalanx ratios, and benchmark devices.

To assess robustness against the joint coupling assumption, all single-objective optimizations were repeated with a reduced coupling coefficient (κ=0.7). The qualitative trends remain unchanged. The grasp capability $f_{1}$ still favors a short middle and long distal phalanx. The key-press range $f_{3}$ still favors a short proximal and long distal phalanx. The kinematic dexterity $f_{2}$ still favors a long proximal phalanx. The only quantitative difference is that the optimal $l_{3}$ for $f_{2}$ shifts upward from 16.03 mm (κ=1.0) to 17.67 mm (κ=0.7), reflecting the altered Jacobian column weighting. Hence the hierarchical selection strategy remains valid over the physiologically plausible range of coupling ratios, roughly 0.6 to 1.0.

Several limitations point toward future work. The current model is single-finger, rigid-link, and quasi-static. Multi-finger coordination, compliance, under actuation, and dynamic effects such as impact, inertia, and tendon friction are not yet incorporated. The fixed total length of 81 mm is anthropomorphically motivated, but the influence of absolute scale on the trade-offs among $f_{1}$, $f_{2}$, and $f_{3}$ remains unexplored. As noted above, the planar workspace analysis is most suitable for objects with a dominant planar interaction; extending to 3D is an important future direction. Although typing provides a clean, quantifiable testbed, the framework is general and can be adapted to other fine manipulation tasks, for example button pressing or touchscreen operation, by redefining or augmenting $f_{3}$. The current experimental validation is limited to a single design at a fixed speed, without comparison to other phalanx ratios or existing robotic hands; such comparative experiments are needed to fully assess the practical advantages of the proposed optimization framework. Finally, real-time task recognition and adaptive weighting, which allow a finger to dynamically prioritize different objectives, represent an exciting convergence of design optimization and control. It is important to clarify that this is a forward-looking concept, not part of the present work. In future implementations, the finger could physically adapt its effective dimensions (e.g., via telescoping links, variable stiffness materials, or reconfigurable tendon routing) in response to perceived task demands, rather than merely adjusting control parameters within a fixed hardware configuration. Such adaptability would move beyond static design optimization toward an integrated design-control framework. Despite these limitations, the proposed methodology offers a principled, weight-free, and reproducible template for multi-objective dimensioning of tendon-driven robotic fingers under a strict length budget. It combines workspace partitioning, global dexterity averaging, and Pareto-based hierarchical selection.

## 5. Conclusions

This paper presented a Pareto-based multi-objective optimization framework to determine the optimal phalanx lengths of a biomimetic finger subject to a fixed total-length constraint. Three performance criteria—grasp capability (covering both power and precision modes), kinematic dexterity, and key-press reachability—were simultaneously evaluated through rigorous parametric analysis. The Pareto analysis revealed substantial trade-offs among these objectives: kinematic dexterity and key-press range are most strongly competing (*r* = −0.955), while grasp capability and kinematic dexterity show a moderate negative correlation (*r* = −0.439), while key-press reachability proved the most sensitive to dimensional variations, exhibiting a 24.7% spread across the design space. These findings underscore that no single phalanx proportion can simultaneously maximize all performance indices, motivating the need for principled compromise strategies.

To navigate these trade-offs, a hierarchical selection strategy was employed to identify a recommended design ($l_{1}=40.44$ mm, $l_{2}=21.39$ mm, $l_{3}=19.17$ mm) that achieves 98.0% of the maximum key-press range while maintaining $f_{1}$ within 1.2% of its maximum and preserving competitive $f_{2}$ performance without significant degradation relative to the extreme $f_{3}$-maximizing design. Complementary design guidelines were distilled from extensive parametric studies: a shorter middle phalanx expands the shared workspace region critical to dual grasp modes, while judicious phalanx proportioning improves the average reciprocal condition number, indicating enhanced kinematic isotropy over the sampled posture set. Furthermore, sensitivity analysis with a reduced tendon coupling coefficient (κ = 0.7) confirmed that the observed optimization trends and qualitative design guidelines remain valid, attesting to the robustness of the conclusions across variations in actuation routing. Experimental validation of the recommended compromise design demonstrated a 98.8% cross-row keystroke success rate at approximately 5.5 Hz over 1000 consecutive trials, establishing the practical feasibility of the Pareto-optimal solution under demanding high-speed repetitive operation.

Several limitations of the present study should be acknowledged. First, the analysis is confined to a single finger with rigid-link kinematics, precluding investigation of whole-hand coordination and compliant mechanisms. Second, experimental validation is currently limited to the key-press reachability criterion; the grasp capability and dexterity objectives have not yet been physically tested. Third, the optimization assumes quasi-static conditions and does not account for dynamic effects such as impact during key press or inertial loading during rapid motion. Fourth, the keyboard typing experiment was carried out using only the recommended compromise design at a single speed (≈5.5 Hz), without comparison against other phalanx length ratios, different typing speeds, or existing robotic hands. Therefore, the relative superiority of the recommended design over alternative configurations or benchmark devices remains to be experimentally verified.

Looking ahead, future research will extend the framework to multi-finger coordinated manipulation, incorporate compliant joint designs to exploit intrinsic passive adaptability, and explore dynamic task scenarios where inertial and contact forces become significant. Experimental characterization of grasp quality and dexterity for the full Pareto set—including $f_{1}$- and $f_{2}$-dominant configurations—will be essential to complete the empirical validation. Comparative studies evaluating different phalanx ratios, varying operating speeds, and benchmarking against existing robotic hands are also needed to fully demonstrate the practical advantages of the proposed optimization framework. The methodology developed here provides a general template for multi-objective dimensioning of tendon-driven robotic fingers, offering a principled pathway to balance competing functional requirements through quantitative trade-off analysis.

## Figures and Tables

**Table 1:** Results of keystroke reliability verification.

Key	Test Count	Success Count	Success Rate
R	200	199	99.5%
T	200	197	98.5%
Y	200	199	99.5%
F	200	195	97.5%
G	200	198	99%
Total	1000	988	98.8%

**Table 2:** Comparison of single-objective optima under *κ* = 1 and *κ* = 0.7.

Objective	k	$l_{1}$	$l_{2}$	$l_{3}$	$f_{k}\;Value$	Best Trends
$f_{1}$	1.0	42.03	19.00	19.97	905.19	$l_{2}$ ↓, $l_{3}$ ↑
$f_{1}$	0.7	42.53	19.00	19.47	922.10	$l_{2}$ ↓, $l_{3}$ ↑
$f_{2}$	1.0	44.00	20.97	16.03	0.2477	$l_{1}$ ↑, $l_{3}$ ↓
$f_{2}$	0.7	44.00	19.33	17.67	0.2281	$l_{1}$ ↑
$f_{3}$	1.0	40.00	21.03	19.97	33.07	$l_{1}$ ↓, $l_{3}$ ↑
$f_{3}$	0.7	40.10	20.95	19.95	37.86	$l_{1}$ ↓, $l_{3}$ ↑

## Data Availability

The original contributions presented in this study are included in the article and its Supplementary Materials. Further inquiries can be directed to the corresponding author.

## Supplementary Materials

The following supporting information can be downloaded at https://www.mdpi.com/article/10.3390/biomimetics11080529/s1, Video S1: Key-press experimental demonstration.
